# Stress fiber anisotropy contributes to force-mode dependent chromatin stretching and gene upregulation in living cells

**DOI:** 10.1038/s41467-020-18584-5

**Published:** 2020-09-29

**Authors:** Fuxiang Wei, Xiangyu Xu, Cunyu Zhang, Yawen Liao, Baohua Ji, Ning Wang

**Affiliations:** 1 Laboratory for Cellular Biomechanics and Regenerative Medicine, Department of Biomedical Engineering, College of Life Science and Technology, Huazhong University of Science and Technology, Wuhan, 430074 Hubei China; 2 Department of Applied Mechanics, Beijing Institute of Technology, 100081 Beijing, China; 3 Biomechanics and Biomaterials Laboratory, Department of Engineering Mechanics, Zhejiang University, Hangzhou, 310027 Zhejiang China; 4 Department of Mechanical Science and Engineering, The Grainger College of Engineering, University of Illinois at Urbana-Champaign, Urbana, IL 61801 USA

**Keywords:** Biological techniques, Computational biophysics

## Abstract

Living cells and tissues experience various complex modes of forces that are important in physiology and disease. However, how different force modes impact gene expression is elusive. Here we apply local forces of different modes via a magnetic bead bound to the integrins on a cell and quantified cell stiffness, chromatin deformation, and *DHFR* (dihydrofolate reductase) gene transcription. In-plane stresses result in lower cell stiffness than out-of-plane stresses that lead to bead rolling along the cell long axis (i.e., alignment of actin stress fibers) or at different angles (90° or 45°). However, chromatin stretching and ensuing *DHFR* gene upregulation by the in-plane mode are similar to those induced by the 45° stress mode. Disrupting stress fibers abolishes differences in cell stiffness, chromatin stretching, and *DHFR* gene upregulation under different force modes and inhibiting myosin II decreases cell stiffness, chromatin deformation, and gene upregulation. Theoretical modeling using discrete anisotropic stress fibers recapitulates experimental results and reveals underlying mechanisms of force-mode dependence. Our findings suggest that forces impact biological responses of living cells such as gene transcription via previously underappreciated means.

## Abstract (web summary)

Living cells and tissues experience various complex modes of forces but how different force modes impact gene expression is elusive. Here authors apply forces via magnetic beads to integrins on a cell surface and observe force-mode dependent chromatin stretching and gene upregulation in cells and identify underlying mechanisms.

## Introduction

It is well established that living cells and tissues respond to mechanical force stimulation such as shear stresses in the blood or interstitial flow^1,2^, tractional forces at cell–matrix contacts^3^, and cell–cell contacts^4^. Mechanical properties of the living cells and tissues are also known to be critical in regulating their biological responses in physiology and diseases^5–8^. However, how different modes of forces impact gene expression is elusive. For example, while it is well known that shear stresses at the cell apical surface produce different signals and cellular effects than do stretching forces at the base or sides of endothelial cells, as vessel diameter changes during dilation or constriction^9,10^, the underlying mechanisms of how gene transcription is altered by various force modes remain unclear. There are only a few well characterized technologies that can apply controlled local mechanical forces to a single living cell. One such method is the atomic force microscopy (AFM) that in general applies an indentation force on the apical surface of the cell^11^. Another method is the optical tweezer that uses a focused laser light to trap a micrometer-sized particle to apply a lateral force on the cell apical surface^12^. The third method is the method of magnetic twisting cytometry (MTC) or magnetic gradient pullers. In magnetic gradient pullers, an electromagnetic inhomogeneous field is generated at the tip of the tweezer to pull on a magnetic bead attached to the apical surface of the cell^13^. In MTC, a ferromagnetic bead is magnetized with a strong magnetic field pulse in the horizontal direction (e.g., along *Y*-axis) and then a weak homogenous magnetic field is applied in the vertical direction (along *Z*-axis) to rotate the bead about the *X*-axis^14,15^. This early version of the MTC can only rotate the magnetic bead about one axis—the *X*-axis and is thus called one dimensional (1D) MTC. Because of the technical difficulty to compare mechanical responses of the living cell to various mechanical probes^16^, it is not clear how surface force modes influence cellular responses and functions. Here we describe a strategy of using the three-dimensional MTC (3D MTC)^17,18^ to apply forces in any desired directions to the same living cell by rotating the magnetic bead about any axes. We find that for a given stress amplitude, a living cell responds to a local in-plane stress differently from responding to a local out-of-plane complex stress by stretching chromatin and upregulating gene transcription to different levels. Experimental results in living cells and theoretical modeling analyses using discrete anisotropic elements reveal that stress fiber anisotropy determines force-mode dependent cell stiffness and chromatin stretching and thus regulates gene upregulation.

## Results

### A strategy of applying local in-plane stress

**Fig. 1:**
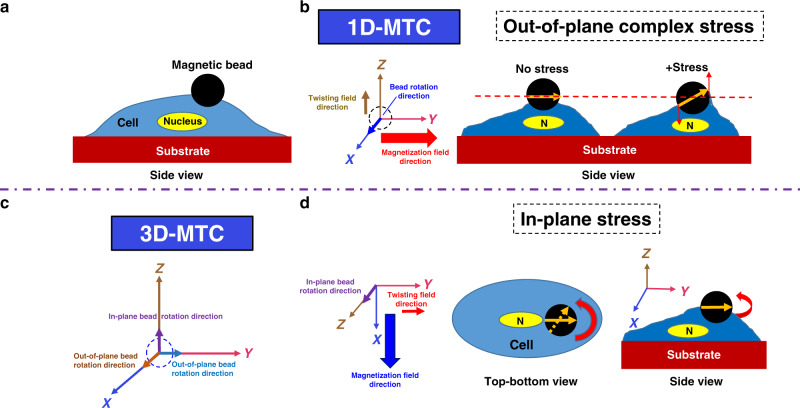
Strategies of applying different stress modes to a cell. **a** Schematic of the cell (blue color) with its nucleus (yellow color); the big black dot was the ferromagnetic bead. The cell was placed in a culture dish in the center of the coils and the focal plane of the microscope. Not drawn to scale. **b** Schematic of one dimensional magnetic twisting device (1D MTC) to apply force on the cell. The bead rotated in the *Y*–*Z* plane about the *X*-axis (torque was in the *x* direction) and generated a local complex stress. **c** Schematic of three-dimensional magnetic twisting device (3D MTC) to apply force on the cell. The bead rotated in the *X*–*Z* plane (out-of-plane) about the *Y*-axis (the right-hand rule), the *Y*–*Z* plane (out-of-plane) about the *X*-axis, or the *X*–*Y* plane (in-plane) about the *Z*-axis. **d** Schematic of magnetizing the magnetic bead in the *X* direction and twisting it in the *Y* direction. The bead rotated in the *X*–*Y* plane (about the *Z*-axis) which was parallel to the substrate surface of cell spreading and generated a local stress.

The 1D MTC generated a bead rotation that was out-of-the *X*–*Y* plane and thus generated a complex stress on the cell surface: as the bead rotated in the *Y*–*Z* plane, the bead edge that moved upward stretched the cell membrane and the bead edge that moved downward compressed the cell membrane (Fig. 1a, b). In contrast, the 3D MTC could magnetize a magnetic bead in any desired direction^17,18^ (*X*, *Y*, or *Z*) and applied a homogeneous twisting field in any desired direction (*X*, *Y*, or *Z*) (Fig. 1c). When a magnetic bead was magnetized in *Z* direction and then twisted in the *Y* direction or in the *X* direction, an out-of-plane complex stress was applied as the bead rotated about the *X*-axis or the *Y*-axis, respectively (Fig. 1c). Here we describe a strategy to apply local in-plane stresses with the 3D MTC: when a magnetic bead was magnetized in *X* direction and then twisted in *Y* direction (Fig. 1d, left), a local stress was applied to the cell surface as the bead rotated about the *Z*-axis (Fig. 1d, middle, right).

### Cell stiffness and chromatin stretching under different stress modes

**Fig. 2:**
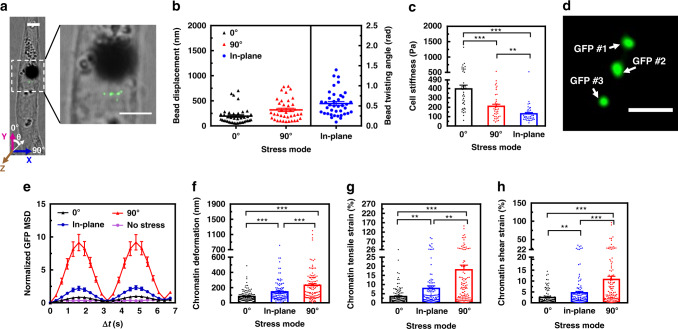
Mechanical anisotropy of the cell and of the chromatin induced by different force modes. **a** A representative image of adherent and elongated CHO (Chinese hamster ovary) cell with the GFP labeled chromatin domain (green dots; see the enlarged image within the dashed white lines). A 4-μm RGD-coated ferromagnetic bead (the solid black ball) was attached to the cell surface via integrins. Theta represents the angle of bead rolling direction with respect to the long axis of the cell (this notation applies to all cells in all figures). The bead and GFP spots in the white box were enlarged and shown on the right. Scale bar, 3 μm. The in-plane (*X*–*Y* plane) rotation has a direction along the *Z*-axis and the two out-of-plane rotations have directions along the *X*- and *Y*-axes, using the right-hand rule (see Fig. 1). **b** Peak 2D displacements of the center of the magnetic bead in the *X*–*Y* plane at stress angles 0° or 90° for the out-of-plane stress mode or the peak bead twisting angles for the in-plane stress mode in the same cell. In all stress modes, the peak amplitudes of the sinusoidal magnetic fields were maintained at 15 Pa and 0.3 Hz. Each triangle or dot represents one cell. **c** Cell stiffness computed with different stress modes. *P* = 0.0079 between 90° and in-plane stress modes; *P* < 0.001 between 0° and 90°, 0° and in-plane stress modes. **d** Fluorescent image of the three GFP spots in the same chromatin of a representative cell. Scale bar, 1 μm. **e** Normalized mean squared displacement (MSD) of all individual GFP spots when the stress (15 Pa at 0.3 Hz) was applied at 0° or 90° or in-plane mode. No stress data represent the spontaneous GFR spots movements in the absence of force application. **f** Chromatin deformation (i.e., changes of distances between any two GFP spots in the same chromatin domain) depends on stress modes. *P* < 0.001 between each different stress modes. **g** Tensile strains of the chromatin were computed from chromatin deformation^21^. *P* = 0.0055 between 0° and in-plane stress modes; *P* = 0.0017 between in-plane and 90° stress modes; *P* < 0.001 between 0° and 90° stress modes. **h** Shear strains were computed from the same chromatin deformation. *P* = 0.00701 between 0° and in-plane stress modes; *P* < 0.001 between in-plane and 90° and 0° and 90° stress modes. For **b**, **c**, and **e–h**, mean ± s.e.m.; *n* = 39 cells, 29 independent experiments; **P < 0.01; ***P < 0.001. *P* values were calculated and corrected using two-tailed Student’s *t*-test and Bonferroni correction. Source data are provided as a Source data file.

Using the strategy of in-plane stress, we applied local stresses to the cell surface via specific receptors like integrins using an Arg-Lyn-Asp (RGD) peptides coated magnetic bead (Fig. 2a). The peak amplitude of the sinusoidal wave was kept at 15 Pa and loading frequency was kept at 0.3 Hz. We measured the bead rotation angles (radians) when the in-plane stress was applied (Fig. 2b). We also measured 2D projection of center displacements of the bead in the *X*–*Y* plane on the surface of the same cell as the bead rolled either along the long axis of the cell (0° stress mode) or transverse the long axis of the cell (90° stress mode) when the out-of-plane complex stress was applied (Fig. 2b). As expected from the anisotropic mechanical behaviors of the living cell^17^, the bead displacements were much less for the 0° mode (i.e., along the long axis of the cell and thus the direction of most stress fibers) than for the 90° mode (Fig. 2b). Using the published method of computing cell stiffness by taking into account the bead–cell contact area^19^, we computed cell stiffness of the same living cell under the condition of different stress modes. Cell stiffness was twice as much for the 0° mode as for the 90° mode (Fig. 2c). Interestingly, cell stiffness was lowest when the in-plane stress was applied (Fig. 2c). To determine how the local surface stresses deform the chromatin, we quantified deformation of chromatin domains where green fluorescent protein (GFP) labeled transgene *DHFR* (dihydrofolate reductase) resided^20^ (Fig. 2d). Mean Square Displacements (MSDs) of the GFP spots (Fig. 2e) and the changes in distances between any two GFP spots (chromatin deformation) (Fig. 2f) were highest for the 90° stress mode, intermediate for the in-plane stress mode, and lowest for the 0° mode. We then computed stretching of the chromatin domain containing the *DHFR* gene by quantifying the tensile strains and the shear strains of the chromatin^21^ and found that the in-plane stress mode resulted in the strains that were higher than the 0° mode and lower than the 90° mode (Fig. 2g, h). The data showed that tensile strains were about twice as much as the shear strains, suggesting that the dominant form of the chromatin deformation was tensile (i.e., stretching) for the in-plane mode. This result was unexpected. Since in contrast to the out-of-plane stress modes that resulted in predominantly normal strains for the 0° mode and similar magnitudes of normal and shear strains for the 90° mode at the cell cortex, the in-plane stress mode caused mostly shear strains at the cell cortex (Supplementary Fig. 1; Supplementary Table 1), but inside the nucleus the chromatin domain deformation via the in-plane mode was mainly tensile, suggesting that the complex structural arrangements of the cytoskeleton, linker of nucleoskeleton and cytoskeleton, and the nuclear lamins propagate the surface stress into the nucleus as a complex stress to result in mainly tensile deformation in the chromatin. Next we examined how stress amplitudes of the in-plane mode would impact chromatin deformation and cell stiffness. MSDs of individual GFP spots increased with the stress amplitude (Supplementary Fig. 2a–d). The bead twisting angle increased linearly with stress amplitude within the range of stress that we had applied (Supplementary Fig. 2e). As a result, cell stiffness did not change with the amplitude of the in-plane stress (Supplementary Fig. 2f). Furthermore, chromatin deformation increased with the in-plane stress amplitude (Supplementary Fig. 2g), suggesting that the structural changes in the chromatin domain are direct mechanical responses. Together these data show that in-plane stress induces different responses on cellular mechanical properties (i.e., cell stiffness) and chromatin deformation from the out-of-plane complex stresses.

### Gene upregulation varies with stress modes

**Fig. 3:**
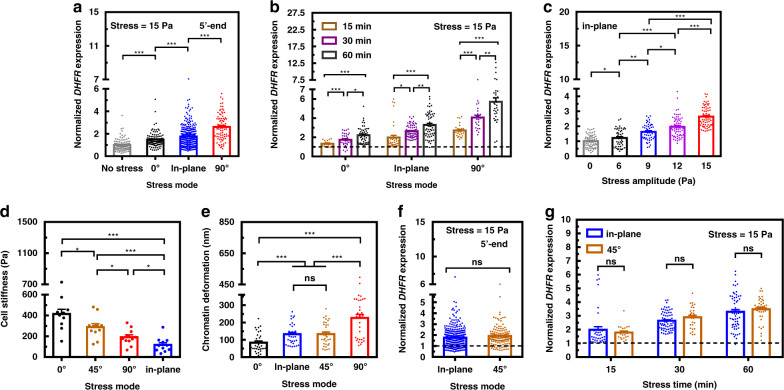
Transcription varies with stress modes and increases with stress duration. **a** Summarized data of *DHFR* transcription detected with 5′-end probes. Stress was applied to each cell only once at one particular angle for all FISH experiments. Fluorescently labeled 5′-end probes were used to detect expression of the first 1700 bps *DHFR* mRNA. Controls (No stress) were the cells in the same dish without attachment of magnetic beads. The stress was applied for 2 min at 15 Pa and 0.3 Hz. *P* < 0.001 between each different stress modes. Mean ± s.e.m.; *n* = 115, 97, 344, and 85 cells for no stress, 0°, in-plane, and 90° stress modes, respectively, in nine separate experiments; ***P < 0.001. **b** *DHFR* transcription upregulation depends on stress (15 Pa at 0.3 Hz) duration for both in-plane and out-of-plane modes. *P* = 0.016 between 30 and 60 min under 0° stress mode; *P* = 0.025 between 15 and 30 min and *P* = 0.0015 between 30 and 60 min under in-plane stress mode; *P* = 0.0039 between 30 and 60 min under 90° stress mode; *P* < 0.001 between other conditions under each stress mode. Mean ± s.e.m.; 0°: *n* = 21, 33, and 52 cells at 15, 30, and 60 min; in-plane: *n* = 38, 66, and 60 cells at 15, 30, and 60 min; 90°: *n* = 26, 25, and 32 cells at 15, 30, and 60 min. Three independent experiments. *P < 0.05; **P < 0.01; ***P < 0.001. The dashed line was the no stress control. **c** *DHFR* gene upregulation depends on stress amplitudes of the in-plane mode. All stresses were applied at 0.3 Hz for 30 min. *n* = 71 cells for 0 stress; *n* = 47, 50, 68, and 66 cells at 6, 9, 12, and 15 Pa, respectively. *P* = 0.032 between 0 stress and 6 Pa; *P* = 0.0033 between 6 and 9 Pa; *P* = 0.014 between 9 and 12 Pa; *P* < 0.001 between other different stress amplitudes. Mean ± s.e.m.; *P < 0.05; **P < 0.01; ***P < 0.001. **d** Cell stiffness computed from different stress modes. *P* = 0.036 between 0° and 45° stress modes; *P* = 0.039 between 45° and 90° stress modes; *P* = 0.037 between 90° and in-plane stress modes*; P* < 0.001 between 0° and in-plane stress modes as well as 45° and in-plane stress modes. Mean ± s.e.m.; *n* = 11 cells, eight independent experiments. *P < 0.05; ***P < 0.001. **e** Chromatin deformation (chromatin deformation, i.e., changes of distance between GPF spots) with different stress modes. No difference was found between the 45° mode and the in-plane mode. *P* = 0.97 between in-plane and 45° stress modes; *P* < 0.001 between other different stress modes. Mean ± s.e.m.; *n* = 33 GFP spots; 11 cells, eight independent experiments. ***P < 0.001; ns = not significantly different. **f** Comparison of *DHFR* upregulation (quantified with 5′-end probes) between the out-of-plane 45° mode and the in-plane mode. The stress was applied for 2 min at 15 Pa and 0.3 Hz. *P* = 0.069 between in-plane and 45° stress modes. Mean ± s.e.m.; *n* = 344 and 140 cells for in-plane and 45° stress modes; nine separate experiments; ns = not significantly different. The dashed line was the baseline transcription level in the absence of force. **g** *DHFR* upregulation was similar at longer durations of stress (15 Pa and 0.3 Hz) between the 45° out-plane mode and the in-plane mode. *P* = 0.44, 0.14, and 0.4 between in-plane and 45° stress modes under 15, 30, and 60 min, respectively. Mean ± s.e.m.; 45°: *n* = 21, 29, and 39 cells at 15, 30, and 60 min, respectively; in-plane: *n* = 38, 66, and 60 cells at 15, 30, and 60 min, respectively. Three independent experiments; ns = not significantly different. The dashed line was the baseline transcription level in the absence of force. *P* values were calculated and corrected using two-tailed Student’s *t*-test and Bonferroni correction. Source data are provided as a Source data file.

Next we examined how *DHFR* gene transcription might change in response to different stress modes at the same stress amplitude and frequency. To minimize contributions of potential confounding factors in the cytoplasm to *DHFR* transcription, we employed a published strategy of using fluorescently labeled 5′-end probes that could detect the transcription of the first 1700 bps *DHFR* mRNA^20^. This way we could detect processes of transcription by reading the partial transcripts as early as seconds to a few minutes before the full transcript of *DHFR* was completed (*DHFR* gene is 34 kb long and it takes ~10 min to complete one transcript). When the stress was applied at 15 Pa and 0.3 Hz for 2 min, *DHFR* transcription was upregulated from the baseline: the in-plane stress mode caused an upregulation that was higher than the 0° mode and lower than the 90° mode (Fig. 3a). Extending the duration of stress application to 60 min, we found that all three stress modes resulted in a time-dependent elevation of transcription of *DHFR* (Fig. 3b). In line with the published results, the in-plane mode also led to a stress-amplitude dependent elevation of *DHFR* transcription (Fig. 3c). Since the extent of the chromatin deformation and *DHFR* upregulation under the in-plane mode was between those under the 0° mode and the 90° mode, we wondered if the 45° stress mode (i.e., the bead rotation was along the direction that was diagonal between the long axis and the short axis of the cell) would cause similar responses from the cell as the in-plane mode. It is interesting that although cell stiffness probed via the 45° out-of-plane mode was ~3 times that via the in-plane mode (Fig. 3d), the chromatin deformations were very similar (Fig. 3e). In addition, there was no difference in *DHFR* transcription upregulation between the 45° mode and the in-plane mode, either after 2 min stress or after longer stress application (Fig. 3f, g), suggesting that although these two modes are quite distinct in cell surface deformation (one is out-of-plane and the other is in-plane) that led to different stiffness values of the same cell, their impacts on the chromatin deformation and *DHFR* transcription were similar.

### Stress fiber anisotropy determines chromatin deformation and gene upregulation

**Fig. 4:**
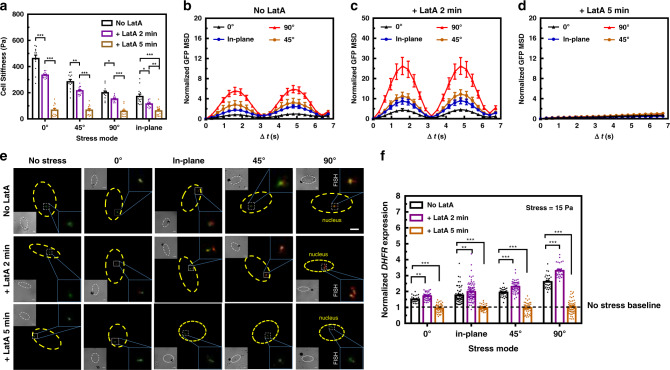
Actin stress fibers dominate cytoskeletal anisotropy-dependent cellular responses. **a** Cell stiffness under different stress modes before adding Latrunculin A (No LatA control), after adding Latrunculin A (1 μM) for 2 min (+LatA 2 min) or 5 min (+LatA 5 min). *P* = 0.0075 between No LatA and +LatA 2 min under 45° stress mode; *P* = 0.023 between No LatA and +LatA 2 min under 90° stress mode; *P* = 0.039 between No LatA and +LatA 2 min and *P* = 0.0032 between +LatA 2 min and +LatA 5 min under in-plane stress mode; *P* < 0.001 between other conditions under each stress mode. Mean ± s.e.m.; *n* = 12 cells; nine independent experiments. *P < 0.05; **P < 0.01; ***P < 0.001. Normalized mean squared displacement (MSD) of all individual GFP spots when the stress (15 Pa at 0.3 Hz) was applied at 0°, 45°, or 90° out-of-plane mode or in-plane mode before adding LatA (**b**), after adding LatA for 2 min (**c**), or for 5 min (**d**). Mean ± s.e.m.; *n* = 12 cells, nine independent experiments. **e** Representative images of RNA FISH at no stress, 0°, 45°, or 90° out-of-plane mode or in-plane mode. Stress (15 Pa at 0.3 Hz) was applied before adding Latrunculin A (No LatA control), after adding Latrunculin A for 2 min (+LatA 2 min), or 5 min (+LatA 5 min). The yellow dashed lines highlighted the area of the nucleus. The GFP chromatin domains (green color) and the *DHFR* FISH fluorescence (red color) in the white box of each image were enlarged and shown on the right. The brightfield image of each cell was shown in an inset at the left site and the scale bar was 3 μm within each inset. The black dot was the magnetic bead attached to the cell surface. The nucleus was highlighted with a dashed line. The scale bar on the top right panel was 3 μm. **f** *DHFR* transcription varies with duration of LatA treatment for both in-plane and out-of-plane modes. *P* = 0.0023 between No LatA and +LatA 2 min under 0° stress mode; *P* = 0.0048 between No LatA and +LatA 2 min under in-plane stress mode; *P* < 0.001 between other conditions under each stress mode. Mean ± s.e.m.; 0°: *n* = 38, 45, and 47 cells; in-plane: *n* = 58, 89, and 41 cells; 45°: *n* = 35, 52, and 40 cells; 90°: *n* = 28, 31, and 62 cells for No LatA control, +LatA 2 min and +LatA 5 min, respectively; three independent experiments. Control was the cells applied with the same stress but not treated with LatA. **P < 0.01; ***P < 0.001. The dashed line indicated the *DHFR* gene expression levels without applying the stress. *P* values were calculated and corrected using two-tailed Student’s *t*-test and Bonferroni correction. Source data are provided as a Source data file.

Next we set out to determine what dominates anisotropy of cell stiffness, chromatin deformation, and gene transcription upregulation. Since filamentous actin (F-actin) is a major player in cell stiffness^22^, we used a specific F-actin inhibitor, Latrunculin A (LatA), to treat the cells for various times to disrupt actin stress fibers. Actin stress fibers number decreased when the cells were treated with LatA for 2 min; stress fibers became short, disorganized, aggregated structures after 5 min LatA treatment in the absence of obvious cell rounding (Supplementary Fig. 3a–c). As a result, cell stiffness decreased gradually at 2 min for all stress modes; by 5 min of LatA treatment, the differences in cell stiffness between various stress modes disappeared (Fig. 4a). The cell stiffness data are consistent with the loss of structural integrity of the stress fibers. Interestingly, MSDs of chromatin GFP spots, a measure of chromatin deformation, increased after 2 min of LatA treatment (compare Fig. 4c with Fig. 4b), likely due to the fact that stress fibers were only partially disrupted (Supplementary Fig. 3b) and that the remaining stress fibers were able to transmit forces to the nucleus that was softened by the reduction in the F-actin in the cytoplasm. Five minutes after LatA treatment, however, force-induced chromatin deformation completely disappeared under all stress modes (Fig. 4d), likely because stress fiber integrity and the force-transmission pathway to the chromatin were completely disrupted. Interestingly, the gene upregulation under various stress modes after LatA treatment followed the same trend as the chromatin deformation: 2 min after LatA, transcription was increased when compared to that without LatA treatment for all stress modes and there was anisotropy in changes in transcription under different stress modes; however, no transcription upregulation was observed after 5 min LatA treatment under any stress mode (Fig. 4e, f). Taken together, these results suggest that actin stress fibers are responsible for anisotropy in cell stiffness, chromatin deformation, and rapid gene upregulation under different stress modes.

### Mechanisms of stress-mode dependence of cell stiffness and chromatin deformation

**Fig. 5:**
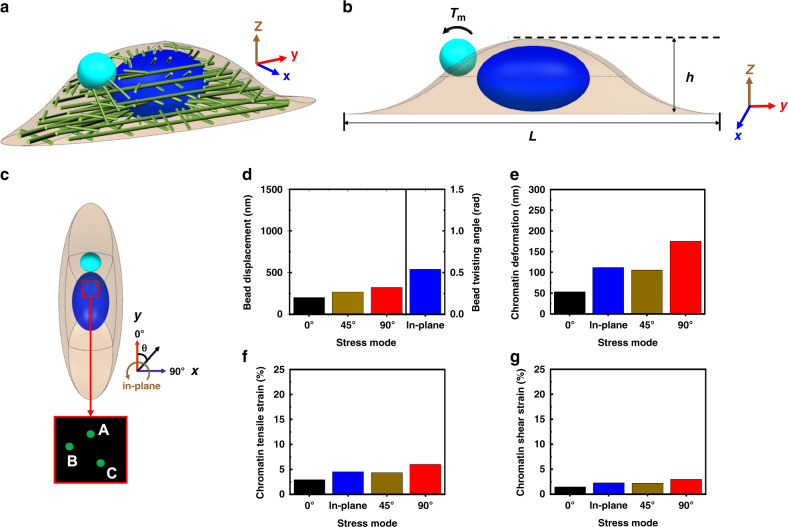
Finite element models (FEM) reveal anisotropic responses of the cell. **a** A 3D illustration of the location of bead and spatial arrangement of the nucleus and actin stress fibers in the FEM model of the cell–bead system: the magnetic bead is in light blue, the nucleus is in dark blue, the cytoplasm is in light brown, and the cell cortex is in brown. Not drawn to scale. **b** The cross-sectional illustration of cell–bead system, showing the dimension of the cell, nucleus, and the bead (see Methods section). The specific torque *T*_m_ (applied stress of 15 Pa) was applied on the cell via the bead. We estimated various parameter values for different components of the cell, obtained from the published report^43^. The Young’s moduli of cell membrane cortex, cytoplasm, and nucleus were 2, 0.25, and 1.0 kPa, respectively; Poisson’s ratios of cell membrane cortex, cytoplasm, and nucleus were 0.3, 0.49, and 0.3, respectively; the actin stress fiber’s stiffness was 20 nN μm^−1^. **c** Illustration of application of load for each stress mode, where theta represents the angle between the rolling direction of the magnetic bead and the long axis of the cell, and the magnified view of the boxed area of nucleus illustrating the three points for calculating the chromatin deformation. **d** Magnetic bead displacements and rotation angle. **e** Chromatin deformation. **f** Chromatin tensile strain. **g** Chromatin shear strain.

To elucidate the underlying mechanism of why the in-plane stress mode leads to the lowest cell stiffness of all the stress modes but induces chromatin deformation comparable to that by the 45° out-of-plane complex stress mode, we employed the approach of finite element model (FEM) (Fig. 5a, b). The FEM of a typical anisotropic cell, whose number of actin stress fibers along the cell long axis was twice as much as the short axis (Fig. 5a), generated stress-mode dependent magnetic bead displacements and chromatin deformation (Fig. 5c–e). The displacement of the magnetic bead along the short axis of the cell (the 90° stress mode) was 1.6 times that along the long axis (the 0° stress mode), and the displacement of the bead at 45° stress mode was in-between these two values (Fig. 5d), indicating that the cell stiffness has distinct anisotropy. In addition, the rotation angle of the magnetic bead under the in-plane stress was ~0.5 rad, suggesting that the cell stiffness induced by the in-plane mode was lower than all out-of-plane stress modes. These results quantitatively reproduced what was measured experimentally in living cells (see Fig. 2 and Supplementary Fig. 4a). We then analyzed the effects of stress modes on chromatin deformation. We used the same approach as the live cell experiments, i.e., three points in the *X*–*Y* plane of a given height inside the nucleus to represent the points in the same chromatin domain (Fig. 5c). We then calculated the change of the distance between each two points before and after loading, and took the average of the three values as the chromatin deformation, with which the tensile and shear strain were calculated. The FEM calculation qualitatively reproduced the live cell experimental measurements (compare Fig. 5e–g with Fig. 2f–h and with Supplementary Fig. 4). Tensile strains and shear strains exhibited anisotropy and the tensile strains were ~twice as large as the shear strains. In addition, the chromatin strains induced by the in-plane stress mode were similar to those by the 45° out-of-plane stress mode.

**Fig. 6:**
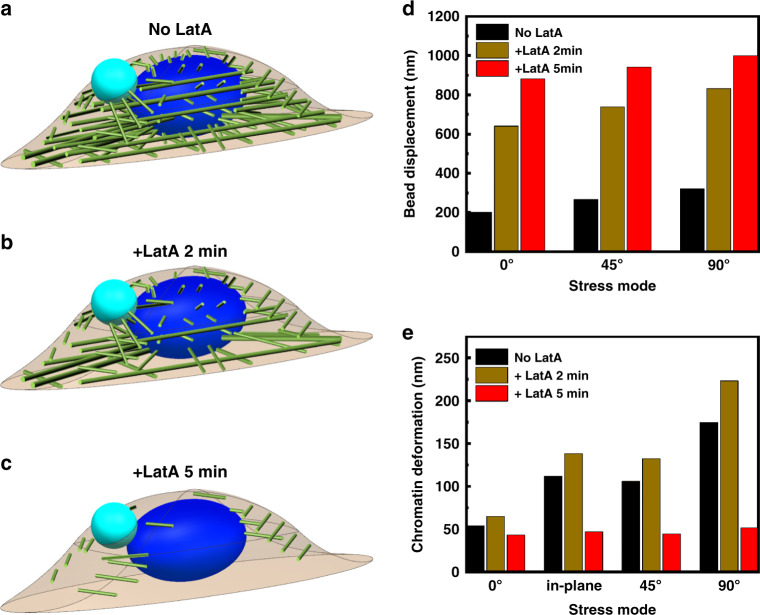
FEM simulation of the effects of disrupting stress fibers on cell and chromatin deformation. **a** No LatA control. **b** +LatA 2 min. **c** +LatA 5 min. Cell shapes and the bead are not drawn to scale. **d** Effects of the LatA treatment on magnetic bead displacements under different stress modes. **e** Effects of LatA treatment on chromatin deformation under different stress modes.

To examine the role of structural integrity of F-actin in cell stiffness anisotropy and chromatin deformation, actin stress fibers were gradually disassembled in the FEM to simulate the inhibitory effect of LatA on F-actin in the live cell experiments. Based on the experimental results from live cells, when treated with LatA for 2 min, actin stress fibers were only partially disassembled, but when treated with LatA for 5 min, the stress fibers were completely destroyed (Fig. 6a–c). The displacements of the magnetic bead after LatA treatment under the three out-of-plane stress modes were shown in Fig. 6d: as stress fibers gradually disassembled, the differences of the bead displacements among the three stress modes were substantially reduced. These results recapitulate the cell experimental results that stress fibers play a key role in the anisotropy of cell stiffness and the average cell stiffness.

It is known from our earlier published reports that the long distance transmission of force into the nucleus depends on actin stress fibers^21,23^. In the FEM, stress fibers between the nucleus and the cell surface were partially (~30%) disrupted after 2 min LatA, leading to weakening the constraints on the nucleus to increase chromatin deformation, which was a result of the elevated stress concentration on the remaining stress fibers to propagate stresses to deform chromatin (Fig. 6e). In contrast, stress fibers between the magnetic bead and the nucleus in the FEM after 5 min LatA treatment were completely disrupted, no longer being able to transfer the load into the nucleus. Hence the chromatin deformation reduced dramatically and the impact of different stress modes on chromatin deformation no longer existed (Fig. 6e). The modeling data therefore are consistent with the experimental results from living cells (see Fig. 4b–d).

Our FEM simulation results showed that under the out-of-plane complex stress modes most of the actin stress fibers were directly stretched or compressed by the rolling motion of the magnetic bead. In contrast, under the in-plane stress mode, most of the stress fibers were not directly stretched. Instead, a tangential deflection was produced which allowed a large rotation of the magnetic bead, resulting the larger extent of cell deformation and thus lower cell stiffness than those by out-of-plane stress modes.

Why chromatin deformation under the in-plane stress mode is similar to that under the 45° out-of-plane stress mode is not obvious. The fact that the two different stress modes (i.e., 45° out-of-plane and in-plane) have similar impacts at a distance, i.e., inside the nucleus, is consistent with the idea that stresses and strains are possibly mediated by the anisotropic cytoskeletal structures and not by a continuous material. To determine the underlying mechanism, we performed additional FEM simulations. Comparing nuclear deformation between the in-plane and 45° stress modes (Supplementary Fig. 5a, b), we found that the nuclear deformation magnitudes and distributions between the two cases were very similar and the nuclear strains near the site of loading were similar as well (Supplementary Fig. 5c, d). In contrast, at the cell cortex near the magnetic bead, the 45° out-of-plane stress mode induced much higher normal strains than the in-plane stress mode whereas the in-plane stress mode induced higher shear strains than the 45° stress mode (Supplementary Fig. 1c, d; Supplementary Table 1). These simulation results suggest that although the cell deformations induced by these two different modes are quite distinct at the cell cortex, the stresses that are propagated into the nucleus by the in-plane and the 45° out-of-plane stress modes are quite similar, possibly due to the fact that all stresses in the cytoplasm have to be concentrated into the nucleus to deform the chromatin, leading to similar chromatin deformation.

## Discussion

**Fig. 7:**
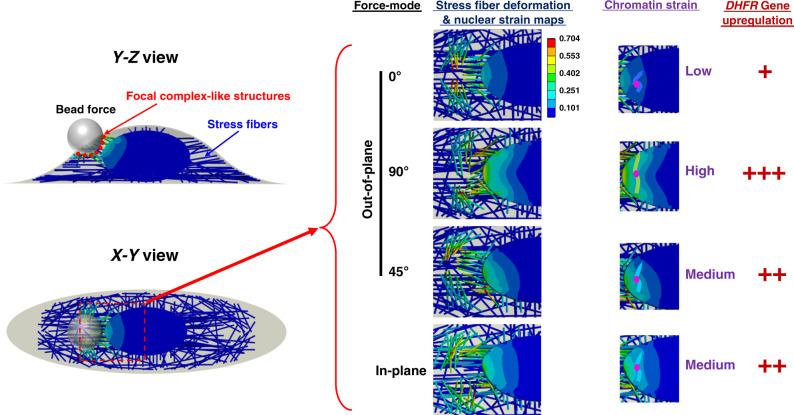
A model of stress fiber anisotropy regulating chromatin strain and gene upregulation. The magnetic bead was bonded to the cell surface via integrins and focal complex-like structures. The schematics on the left were the *Y*–*Z* view and *X*–*Y* view of the bead (4 μm in diameter) and the cell. Each actin stress fiber was modeled as an elastic rod (Young’s modulus was 12 kPa^24^ with 0.4 μm equivalent diameter and Poisson’s ratio was 0.4). There were twice as many stress fibers aligned along the long axis of the cell as those aligned along the short axis of the cell. A constant stress of 15 Pa was applied from the bead to the cell under each force mode. The middle images were the stress fiber deformation and von Mises equivalent strain maps of the nucleus under different force modes. For visual clarity, the bead was removed from each image. The images on the right were chromatin strains under various force modes. Each wiggly line represents a stretched chromatin domain containing the *DHFR* gene locus (the pink dot), which was located at ~2 μm from the edge of the nuclear envelope^37^. From the color scale bar of the nuclear strain and the chromatin domain location, it was estimated that the chromatin domain strain was low (~5%) for the 0° mode (the bead rolling along the cell long axis), high (~25%) for the 90° mode, and medium (~10%) for both the 45° mode and the in-plane mode, comparable with the experimental data from living cells (see Figs. 2 and 3). This model suggests that the extent of chromatin stretching (tensile strain) and thus the level of gene upregulation (the number of the plus sign represents relative levels of gene upregulation) depend on nuclear strain, which is caused by different stress fiber deformation at the nucleus under different force modes. The color scale applies to all images.

In the current study we compared the effects of applying (cell surface) out-of-plane complex stresses with those of applying a (cell surface) in-plane stress on cell mechanical behaviors and biological responses such as gene transcription. We find that there is substantial anisotropy in cell stiffness, chromatin deformation, and gene transcription under different stress modes. For out-of-plane stress modes, rolling the magnetic bead along the short axis of the cell (i.e., 90° from the direction of the most stress fibers) results in larger cell deformation than that along the long axis of the cell (0°) or 45° from the cell long axis. In contrast, applying an in-plane stress leads to more cell deformation than all of the out-of-plane stresses. FEM simulation shows that this is due to the fact that in-plane mode causes little compression or stretching of the stiff actin stress fibers and mainly tangential deflection of the stress fibers, resulting less resistance from the stiff stress fibers. Strikingly, the in-plane stress mode induces similar chromatin deformation as the 45° out-of-plane stress mode in living cells. This result is recapitulated by FEM simulations. To determine why the 45° mode and the in-plane mode induce similar strain maps in the nucleus and how gene transcription is altered by various force modes, we modeled the actin stress fiber as an elastic rod of 12 kPa modulus^24^ and mapped the stress fiber deformation near the nucleus and found that the 0° mode resulted in small stress fiber deformation and low nuclear strain magnitudes; the 90° mode resulted in the large stress fiber deformation and high nuclear strain magnitudes. In contrast, the 45° mode and the in-plane mode led to intermediate stress fiber deformation near the nucleus and medium nuclear strain magnitudes (Fig. 7). These modeling results show that although the 45° mode and the in-plane mode generate different cell cortex strains, the two force modes generate similar stress fiber deformation patterns near the nucleus and thus ensure similar nuclear strain maps, possibly as a result of complex stress distribution in the stress fibers. These modeling data demonstrate that chromatin tensile strain is low for the 0° mode, high for the 90° mode, and medium for both the 45° mode and the in-plane mode, consistent with the experimental data from living cells (see Figs. 2g and 3e). From the current study and a previously published report^20^, it is known that the level of rapid *DHFR* gene upregulation depends on the extent of chromatin stretching or chromatin tensile strain. Together these findings suggest that because the majority of the stress fibers aligns along the long axis of the cell, which results in an anisotropy of cell stiffness, different force modes cause different stress fiber deformation at the nucleus to deform the chromatin and to increase gene expression differently.

Is the out-of-plane complex stress or in-plane stress physiologically relevant modes of force application? It is well known that endothelial cells lining the blood vessels experience fluid shear stresses on their apical surfaces and these stresses are balanced at the basal surfaces at the focal adhesions^25^. In contrast, blood vessels experience blood pressure induced stretching that can cause complex strains in the cells of the vessels along the length of the vessels^26^. It is also known that the myosin-II mediated cellular tractions at the focal adhesions are complex stresses with both in-plane and out-of-plane components^27^. Therefore, exploring the effects of different stress modes on cellular functions is both physiologically relevant and important in understanding how forces impact living cells and tissues.

A living cell’s cytoskeleton consists of three major filament systems: actin filaments, microtubules, and intermediate filaments. The actin filaments are bundled together to form actin stress fibers with myosin-II. We have identified the dominant role of actin stress fibers in anisotropic responses of the cells. Microtubules are known to play mechanical roles in cell functions^28,29^. However, when the cells were treated with colchicine, there were no observable significant changes in cell stiffness or chromatin deformation (Supplementary Fig. 6), suggesting that microtubules play a minor role, if any, in rapid gene upregulation under different stress modes in these CHO cells. Intermediate filaments of the cytoskeleton also play roles in mechanical and biological functions of living cells^30,31^. However, it is known that intermediate filaments play significant roles in cell stiffness only at relatively large deformations^32^. In the future, it will be interesting to explore the potential impact of intermediate filaments in cell responses under different stress modes. CHO cells, generally elongated and have numerous stress fibers after adhesion and spreading, are an epithelial cell line. It remains to be determined in the future whether our findings can be applied to other types of cells.

When the stress fibers are disrupted by LatA, changes in geometry (i.e., bead–cell contact area) and/or bead–cell adhesion might influence cell stiffness measurement. We employed an approach of infecting living cells with talin-GFP, which labeled focal adhesions and focal complex-like structures at the cell basal surface (Supplementary Fig. 7) to examine this possibility. Bead embedding was calculated using the largest diameter of the *X*–*Y* projected image of talin-GFP fluorescence as a measure of the largest bead–cell contact interface; bead–cell contact area was estimated by measuring the *X*–*Y* projected area times the average intensity of the largest fluorescence ring-like structure of talin-GFP surrounding the bead, which was an index of the bead–cell contact area, after taking into account of temporal controls without LatA of laser scanning induced photobleaching (Supplementary Fig. 8a). We found that after 5 min LatA treatment there was only a slight decrease (~10%) both in the bead embedding and the bead–cell contact area from that before the F-actin disruption (Supplementary Fig. 8b, c). This finding suggests that for the same applied torque, the applied stress was increased by ~30% due to the 10% reduction in the contact area, which would be predicted to lead to a ~30% elevation in cell stiffness^19^. However, 5 min after LatA treatment to disrupt stress fibers, the cell stiffness exhibited the opposite trend: the cell stiffness was reduced dramatically for all force modes (see Fig. 4a). These data suggest that the change in cell stiffness after LatA treatment is most likely due to the increase in cell deformation as a result of stress fiber disruption and not due to changes in the bead–cell contact area. On the issue of the adhesion between the RGD-bead and integrins, it is known that a single ligand–integrin bond yielding force is ~100 pN^33^. There are hundreds of integrin receptors that interact with one 4-μm magnetic bead, which is coated with a saturating amount of RGD peptides, suggesting the bonds between the bead and the cell surface are strong. Moreover, we did not observe any bead peeling off the cell surface after stress fiber disruption, suggesting that most bonds are stable. However, it is reported that the characteristic adhesion stress decreases by ~25% after F-actin disruption with cytochalasin D treatment for 5 min^34^. It will be interesting in the future to investigate whether the characteristic adhesion stress changes in various force modes and whether its change after stress fiber disruption impacts on cell stiffness measurement. Furthermore, the stretching distance of the RGD-bead and integrins is only on the order of 0.1–1 nm before the bond is broken^33^, too small to account for the measured bead displacements of several hundred nanometers in live cells (see Fig. 2b). The bead displacements increased by several folds when stress fibers are disrupted by LatA; these changes in bead displacements cannot be explained by the bead–cell bond adhesion alteration. From published articles^14,17,20–22^, it is known that when the RGD-bead is attached to the cell surface, the bead is attached to the cell via the integrin–actin linkages and the bead is tethered by tense F-actin bundles (i.e., stress fibers), providing majority of the resistance to bead rotation (or displacement) under stress. This resistance is an index of cell stiffness. When F-actin bundles are disrupted, this tethering is abolished and hence the resistance to bead rotation (or displacement) under applied stress is substantially reduced and the cell stiffness is dramatically decreased. Together all these suggest that the bead–cell contact area and adhesion play a minor role in cell stiffness after stress fiber disruption.

To better understand the effect of long-range force propagation by the actin stress fibers, we performed simulation by treating the composite of cytoplasm and the actin cytoskeleton as anisotropic uniform elastic materials without explicitly modeling the actin cytoskeleton (denoted as continuous model) (Supplementary Fig. 9). In contrast, in the above analyses, we have employed an FEM (denoted as discrete model, see Fig. 5a) to simulate actin stress fibers as discrete stiff elements embedded in the cytoplasm and calculate their impact on cell and chromatin deformation. The boundary conditions and stress modes in the continuous model are the same as the discrete model. Our results show that although the continuous model could generate similar bead displacements or rotation angles (i.e., similar cell deformation) as the discrete model (compare Supplementary Fig. 9b with 9e) by choosing proper parameter values for the anisotropic properties of the cell, the magnitudes of the chromatin deformation in the continuous model are much smaller than those computed from the discrete model (compare Supplementary Fig. 9c, f), as a result of more localized deformation in the cytoplasm and the nucleus using the continuous model than the discrete model (compare Supplementary Fig. 9a, d). These results are mainly due to the fast decay of the deformation field in the uniform elastic body in the continuous model (stress decays rapidly as the square of the distance), as predicted by the St. Venant’s principle (i.e., a local force only causes local deformation), so that the load from the magnetic bead cannot be effectively transferred to the nucleus. In contrast, the stress fibers in the discrete model can effectively transfer the load to the nucleus to a long distance due to stress concentration, consistent with the previous report of a single stress fiber model^23^. Therefore, the discrete model is more appropriate for simulating long distance force propagation and chromatin deformation of an intact, spread living cell. Besides cytoskeletal filament stiffness, it is known that myosin-II dependent endogenous prestress is critical in the long distance force propagation in the cytoplasm and to the nucleus^20–23,35^. To examine the role of nonmuscle myosin-II, we treated the cells with blebbistatin to inhibit myosin-II. We found that for the same loading, cell stiffness decreased substantially after 50 μM blebbistatin treatment (Supplementary Fig. 10a) and chromatin deformation decreased dramatically (Supplementary Fig. 10b–d), consistent with a finding that myosin-II inhibition reduces cell stiffness^36^ and earlier reports that prestress mediates long force propagation in the cytoskeleton and into the nucleus^21–23,35^. Furthermore, gene upregulation by the applied stress was substantially inhibited when the myosin-II dependent endogenous stress was inhibited with blebbistatin for all force modes (Supplementary Fig. 10e), consistent with and extending the previous finding^20^ when myosin light chain kinase is inhibited by ML-7. In our current discrete FEM model, the impact of the endogenous prestress has been incorporated into the stress fibers to increase the stiffness of the stress fibers and thus is not explicitly shown. Complete disruption of stress fibers in the discrete FEM that removes the influence of myosin-II dependent prestress leads to almost total abolishment of chromatin deformation is consistent with the previous reports^21,23^. Furthermore, it is well established that living cells are viscoelastic and they respond to loading frequencies. However, a recent report finds that loading frequencies between 0.3 and 6 Hz have only modest impacts on chromatin deformation and gene upregulation; the article reveals that force-induced gene upregulation in living cells does not follow the weak power law of rheology but depends on H3K9 demethylation^37^. Although our current discrete FEM is only an elastic model, it has recapitulated the essential features of mechanical and transcriptional responses of living cells at a low loading frequency (0.3 Hz in the current study) and revealed the underlying mechanisms of cellular responses. In the future, the FEM can be extended to include viscoelastic elements in order to better simulate responses under high loading frequencies (e.g., 10–20 Hz). Recently a 3D cell model has been reported to describe the crosstalk among cell adhesions, the cytoskeleton, and the nucleus^38^. It will be interesting to find out if this model can simulate the effects of different stress modes on cell stiffness and direct chromatin stretching.

In summary, we demonstrate that stress fiber anisotropy contributes to force-mode dependent cellular mechanical responses, chromatin deformation, and gene transcription. Our current study is a first attempt to provide insights on better understanding how living cells respond to different modes of complex forces in physiology and disease.

## Methods

### Cell culture and reagents

CHO DG44 DHFR D10 cells (provided by Dr. Andrew Belmont of University of Illinois who created this cell line) were cultured in Ham’s F12 media without thymidine and hypoxanthine (Shanghai Basal) with 10% fetal bovine serum (FBS, Gibco) and 1% Penicillin–Streptomycin (Hyclone)^20^. The authentication of the cell line was confirmed by directly visualizing the GFP-lac repressor staining patterns in these cells. Cells were passaged every 3 days by using TrypLE (Gibco). After centrifuging, cells were dispersed uniformly within 1 mL medium and 200 μL cell suspension was added to a sterile six-pore plate with 3 mL medium which was precoated with 0.1% gelatin (Cat. No. 354236, Corning). Cells were cultured in a 37 °C and 5% CO_2_ incubator for 2 days before experiment. DAPI (4′, 6-diamidino-2-phenylindole) staining of the cell nuclei was constantly performed to monitor these cells during the course of experiments for possible mycoplasma contamination and no signs of mycoplasma contamination were found. Cells in different dishes were randomly assigned during experiments. LatA was purchased from Dalian Meilum Biotech Co., Ltd (Cat. No. J0704A). Colchicine was from MedChem Express Co., Ltd (Cat. No. HY-16569). Blebbistatin was from ApexBio Tech LLC (Cat. No. B1387). F-actin was stained for 4 h before experiment by SiR-actin KIT from Cytoskeleton, Inc. (Cat. No. CY-SC001). Talin-GFP was from Thermo Fisher Scientific (Cat. No. C10611). For talin-F-actin double-staining, actin was labeled by SiR-actin KIT for 4 h after Talin-GFP reagent was added for 16 h. Reagents for RNA FISH: 20× SSC (saline sodium citrate) (Cat. No. AM9763, Thermo Fisher Scientific); 10× PBS (phosphate buffered saline) (Cat. No. AM9625, Thermo Fisher Scientific); BSA (Cat. No. AM2616, Thermo Fisher Scientific); Ribonucleic acid (Cat. No. R1753-2KU, Sigma); Dextran Sulfate Sodium (Cat. No. D8906-10G, Sigma); Deionized Formamide (Cat. No. AM9342, Thermo Fisher Scientific); 16% Formaldehyde (Cat. No. 28906, Thermo Fisher Scientific); Ethanol absolute (Cat. No. 10009218, Sinopharm Co., Ltd). Salmon fibrinogen (Cat. No. SEA-133) and thrombin (SEA-135) were purchased from Pfenex Inc. (CA, USA).

### 3D magnetic twisting cytometry

3D MTC^18^ can generate stresses in any defined direction (*X*, *Y*, *Z*) via rotational movements of ferromagnetic magnetic beads (Boston magnetic beads, purchased from J. Fredberg, Boston, MA) attached to the cell membrane. When the magnetic beads were magnetized in the *Z* direction and applied a homogeneous twisting magnetic field in the *X* or *Y* direction, beads would rotate out of *X*–*Y* plane and move along the short or long axis of the cells. This mode of loading was called the out-of-plane stress mode. When the beads were magnetized in the *X* direction and applied a twisting magnetic field in the *Y* direction (i.e., the beads were magnetized and twisted in the *X*–*Y* plane), the beads would rotate in *X*–*Y* plane and generate stresses. This model of loading was called the in-plane stress mode. In order to apply different modes of force to the same cell, the culture dish containing the cells and the beads were rotated so that the long axis of a cell with the magnetic bead on its surface was aligned along the *Y*-axis. Then the bead was magnetized along the *Z*-axis and a twisting field of 15 Pa was applied along the *Y*-axis. The bead rotation direction vector thus was along the *X*-axis, based on the right-hand rule. This was the 0° out-of-plane mode. The same bead was then re-magnetized along the *Z*-axis and a twisting field of 15 Pa was applied along the *X*-axis. The bead was rotated along the *Y*-axis. This was the 90° out-of-plane mode. The same bead was then re-magnetized along the *Z*-axis again, and simultaneously a twisting field of 10.6 Pa was applied along the *X*-axis and a twisting field of 10.6 Pa was applied along the *Y*-axis, such that the bead rotated at an angle of 45° between the *X*- and *Y*-axes and the sum of the two vector magnitudes was 15 Pa. This was the 45° out-of-plane mode. The in-plane mode was applied by re-magnetizing the same bead along the *X*-axis and applying the twisting field of 15 Pa along the *Y*-axis. Using this strategy, we were able to apply different modes of force of the same amplitude to the same cell and quantify its stiffness and chromatin deformation. For the out-of-plane modes, the bead displacements were measured by quantifying 2D projections of the center of the bead displacements in the *X*–*Y* plane. For the in-plane mode, the bead rotation angle was measured directly by quantifying the 2D rotation angle of the bead edge in the *X*–*Y* plane using the gray pixels of the cell surface right at the bead edge as the reference points. We measured the distance between the bottom of the bead and the rigid cell substrate. The distances were 3.42 ± 0.20 μm (maximum is 5.82 μm, and minimum is 1.32 μm) (Supplementary Fig. 11). If the distance is below 1 μm, the substrate stiffness starts to critically contribute to the apparent cell stiffness measurement. Therefore, the impact of the rigid substrate for our CHO cells in our experimental conditions does not appear to be substantial. Stress applied to cell surface was proportional to the magnitude of the twisting magnetic field. In our experiments, 10, 15, 20, or 25 Gauss magnetic field corresponded to 6, 9, 12, or 15 Pa stress to the cells, calibrated using a viscous fluid standard^14,15^, with which it was determined that the bead magnetic moment constant was 2 Pa Gauss^−1^. The applied stress was equal to the bead magnetic moment constant times the magnetic field divided by 6. One pair of MTC coils could generate 25 Gauss per 100 turns and each pair of the coils in *X*, *Y*, or *Z* direction in the 3D MCT had 180 turns of coils and hence there was a gain of 1.8 in the magnetic field for the coils of the 3D MTC. For example, for a 25 Gauss magnetic field, the stress applied by the 3D MTC was 15 Pa (2 Pa Gauss^−1^ times 25 Gauss times 1.8 divided by 6). To independently calibrate the bead magnetic moment constant, we chose an alternative method by fully embedding the individual magnetic bead in a uniform elastic material made of fibrin gels of known moduli. The salmon fibrinogen was 2 or 4 mg mL^−1^ and 50 μL of fibrinogen was activated by thrombin (1 μL of 100 U mL^−1^) to form the fibrin gels^39^, with the corresponding shear elastic modulus of 60 or 140 Pa, respectively^39^. The stress was applied at 6, 9, 12, or 15 Pa to the bead using the in-plane stress mode at 0.3 Hz and the resultant bead angular rotation (strain) was quantified. The calculated shear elastic modulus of the 2 mg mL^−1^ fibrin gel was 65 Pa and of the 4 mg mL^−1^ fibrin gel was 148 Pa (Supplementary Fig. 12), very close to the published shear modulus values of the fibrin gels^39^. These results suggest that the calibrated bead magnetic moment constant using the viscous standard was accurate and the stiffness measurement by the 3D MTC technology was reliable. Before experiments, the magnetic beads had been coated overnight with a saturating amount (50 μg mL^−1^ RGD per mg of beads) of Arg-Gly-Asp (RGD, PEPTIDE 2000, Cat. No. R4658, Sigma-Aldrich) peptides. Beads and cells were co-incubated for 20 min in the incubator to let the beads tightly attach to the integrins. To ensure there was only one bead per cell in 35 mm petri dish with 18 mm glass-bottomed well (Cat. No. GBD00003-200, Cell E&G), beads were added at a low amount (30 μL of 1 mg mL^−1^ beads) to each dish. Since the chromatin deformation was often very small in the 0° out-of-plane stress mode, we selected the beads whose distances were ~4–5 μm from the GFP spots such that we were able to quantify chromatin deformation at the same location of the same cell under all four stress modes (0°, 45°, and 90° out-of-plane modes and the in-plane mode). The frequency of sinusoidal magnetic field was fixed at 0.3 Hz. Cell stiffness was calculated from bead displacements or rotation angles^19,20^. As the beads might have different degrees of embedding with cells, different embedding coefficients were used to calculate cell stiffness^19^. For the beads whose degree of beads embedding were 36.3% ± 0.7% (*n* = 39 cells, max = 45%, min = 26%) (Supplementary Table 2), embedding coefficients *β* = 0.8, *α* = 0.3 were used to calculate cell stiffness of out-of-plane and in-plane modes, respectively.

### RNA FISH

Custom Stellaris FISH Probes were designed against *DHFR* mRNA (https://www.ncbi.nlm.nih.gov/nuccore/NM_010049.3) by using Stellaris RNA FISH probe Designer (Bioresearch Technologies Inc., Petaluma, CA) to detect the *DHFR* genes transcription. CHO DG44 cells were applied requisite stress amplitude with a certain time, then fixed in 3.7% formaldehyde for 30 min and permeabilized in 70% ethanol for 12 h at 4 °C. Samples were hybridized with *DHFR* Stellaris FISH Probes and incubated in dark humidified incubator for 12 h. Washing 2× in 1 mL wash buffer at 37 °C for 30 min, then proceed to imaging by Leica DMI6000B microscopy after adding 2 mL 2× SSC. Image J was used to analyze the FISH images.

### Microscopy and live cell imaging

Leica DMI6000B with 63 × 1.4 NA oil-immersion objective (Leica, Cat. No. 15506350) was used to image FISH fluorescence via Leica MM AF software. Leica SP8-STED microscopy with 100 × 1.4 NA oil-immersion objective was used to visualize chromatin GFP spots in CHO cells via Leica Application Suite X software. Chromatin deformation was measured following by quantifying changes of distances in the *X*–*Y* plane between chromatin GFP spots in the same chromatin domain^20^. The *Z*-direction displacement was not measured and hence the chromatin deformation and chromatin strains were an underestimate of the total deformation. However, since the CHO cells were very spread (see Fig. 2a), the cell height in *Z*-axis was much less than the dimensions in *X*–*Y* plane, the changes in distances in *Z* was very small. Leica SP8-STED microscopy excited GFP spots and Talin-GFP at 488 nm, SiR-actin at 633 nm. Fluorescence was detected by Hybrid detector.

### Quantification of bead–cell contact area

The CHO cells were cultured for 48 h on glass-bottomed 35-mm dishes, precoated with 0.1% gelatin. Then 20 μL mL^−1^ medium of the reagent of CellLight^®^ Talin-GFP (Thermo, C10611, BacMam 2.0) that was a fusion construct of the c terminus of human talin and emGFP were added to each 35-mm dish for 16 h before experiment. The fusion construct is packaged in the insect virus baculovirus, which does not replicate in mammalian cells, providing accurate and specific targeting to cellular talin-GFP, according to manufacturer’s product description. Bead embedding was calculated using the largest diameter of the *X*–*Y* projected image of talin-GFP fluorescence as a measure of the largest bead–cell contact interface; bead–cell contact area was estimated by measuring the *X*–*Y* projected area times the average intensity of the largest fluorescence ring-like structure of talin-GFP surrounding the bead, which was an index of the bead–cell contact area, after taking into account of temporal controls without LatA of laser scanning induced photobleaching. Adjustment of thresholds was made at the same level for all images to eliminate the background noise. Since bead–cell contact areas varied among different cells, each bead–cell contact area after LatA treatment was normalized by that before the drug treatment.

### Dynamic tracking and image analysis

MATLAB software for data analyzing and image processing^18^ can be downloaded from https://pan.baidu.com/s/1qXCGozy/.

### Finite element model

The FEM of the cell consists of four parts: the cell membrane cortex, the cytoplasm, the nucleus, and the cytoskeleton (Fig. 5a). The first three parts were discretized by 3D bulk elements, while the cytoskeleton, simplified by using contractile actin stress fibers^40^, was discretized by the spring elements^41^. In living CHO cells under the experimental conditions, we found that the cell length was 41.79 ± 0.98 μm, the width was 10.68 ± 0.34 μm, and the height was 8.19 ± 0.17 μm (*n* = 39 cells) (Supplementary Fig. 11). To be consistent with the observed values in live cell experiments, length, width, and thickness of the cell were chosen to be 40, 12, and 8 μm, respectively, and the bead embedding was set to be 38%, as the experimentally measured bead embedding was 36.3% (Supplementary Table 2). The thickness of cell membrane cortex^42^ was set to be 0.25 μm. The nucleus was modeled as an elastic ellipsoid with its long axis as 12 μm and short axis as 7 μm. In order to consider the anisotropic arrangement of the cytoskeletal structure caused by cell polarization, the number of actin stress fibers along the long axis (*Y*-axis) was set to be twice as those along the short axis (*X*-axis). The magnetic bead was also discretized by 3D bulk element, and its adhesion with cell membrane cortex was modeled by bonded contact interactions, which provided the anchor points of the actin cytoskeleton to the bead. The contact of the magnetic bead with the cell surface was chosen to be a few μms from the end of the nucleus. During the loading process, a stable adhesion was assumed between the cell and the substrate, which was regarded as a fixed connection. The mesh was locally refined near the magnetic bead (the bead’s Young’s modulus was assumed to be 2 × 10^5^ MPa), generating a total of 129,543 elements and 208,144 nodes. To determine actin stress fiber deformation near the nucleus, the stress fibers were modeled as elastic rods which had an equivalent diameter of 0.4 μm, whose Poisson’s ratio was 0.4, and whose Young’s modulus was 12 kPa^24^. Other parameters were set to be the same as those used above when stress fibers were modeled as spring elements. von Mises equivalent strains of the nucleus were computed under various force modes.

### Statistical analysis

Two-tailed student’s *t*-test was used for all statistical analyses, except when multiple comparisons were carried out within a given experiment with Bonferroni correction.

## Supplementary information

Supplementary Information

## Data Availability

Data supporting the findings of this paper are available from the corresponding authors upon reasonable request. A reporting summary for this Article is available as a Supplementary information file. Source data are provided with this paper.

## Source data

Source Data

## Code availability

All codes used are available, free of charge, upon request to the corresponding authors. MATLAB software for data analyzing and image processing can be downloaded from https://pan.baidu.com/s/1qXCGozy/. Source data are provided with this paper.
