# Supplementary material for: Stress fiber anisotropy contributes to force-mode dependent chromatin stretching and gene upregulation in living cells
Source: Nat Commun. 2020 Sep 29;11:4902. doi: 10.1038/s41467-020-18584-5 (PMC7524734; doi:10.1038/s41467-020-18584-5)
Supplement: Supplementary file 1 — Supplementary Information [file 41467_2020_18584_MOESM1_ESM.pdf]

Supplementary Information

Stress fiber anisotropy contributes to force-mode dependent chromatin stretching and gene upregulation in living cells

Wei et al.

This file includes Supplementary Figures 1-12 and Supplementary Tables 1 and 2

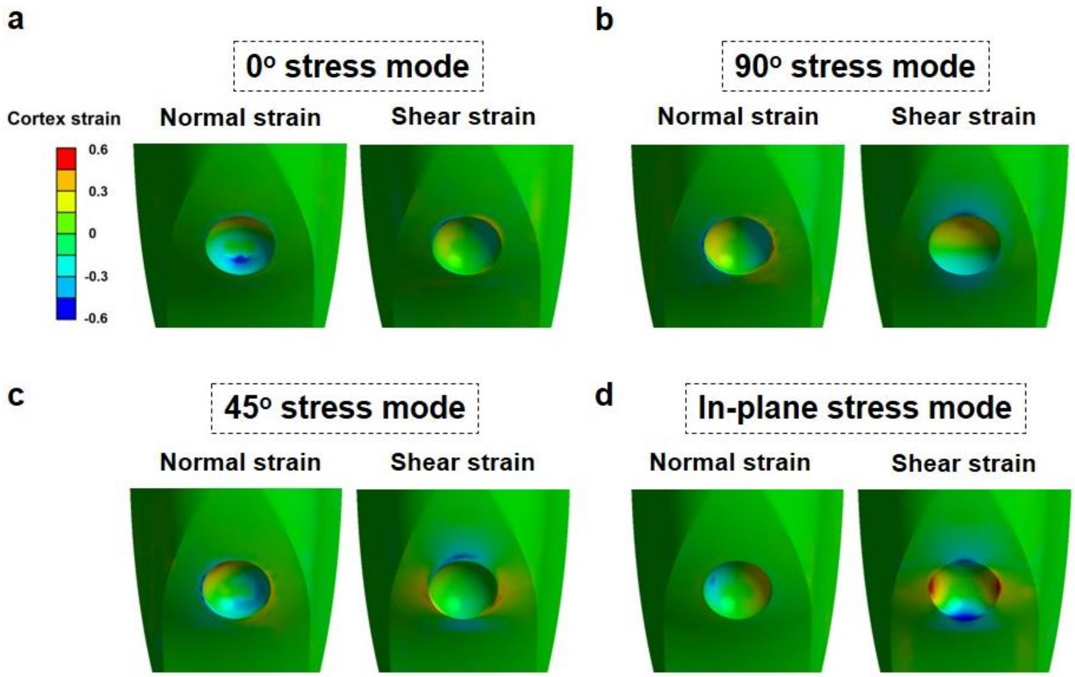

Supplementary Figure 1. Finite element analyses of strain maps at the cell cortex induced by various stress modes. Normal strains and shear strains at the cell cortex (0.25 μm thick) near the magnetic bead under 0 degree (**a**), 90 degrees (**b**), or 45 degrees (**c**) out-of-plane mode or in-plane stress mode (**d**). The ball-like shape at the center of each panel represents the contact area between the magnetic bead and the cell cortex. The color bar applies to (a)-(d). Note that the in-plane stress mode induces much higher maximum shear strains at the cell cortex than each out-of-plane stress mode. For normal strains, positive values represent tensile strains and negative values represent compressive strains; for shear strains, positive values represent clockwise shear and negative values represent counter-clockwise shear.

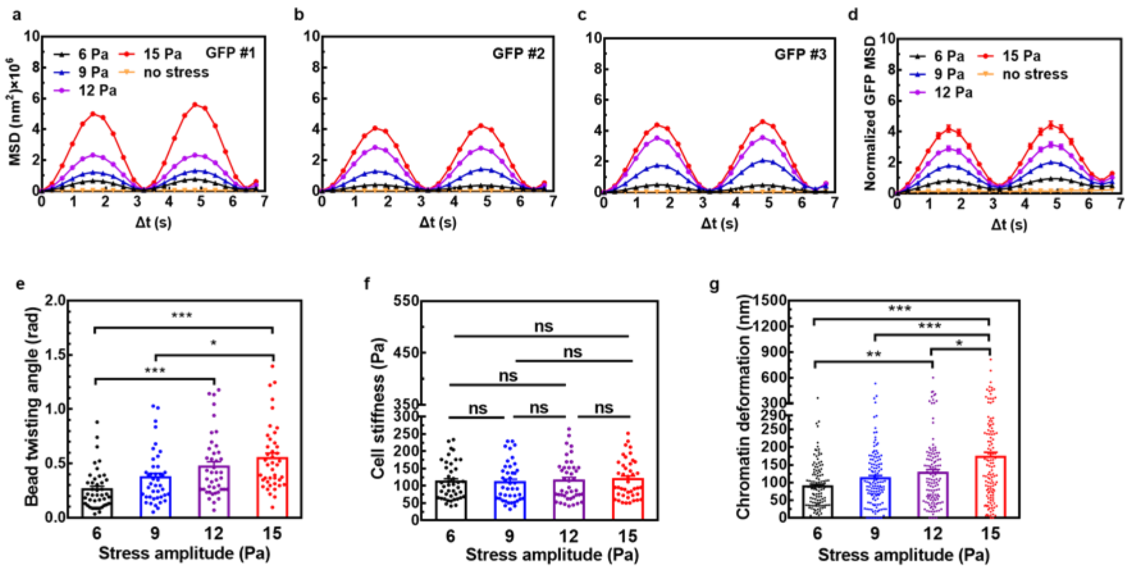

Supplementary Figure 2. In-plane chromatin stretching is stress-amplitude dependent. (**a-c**), Mean squared displacement (MSD) of individual GFP spot 1 (**a**), 2 (**b**), and 3 (**c**) in a representative cell, when the in-plane stress (0.3 Hz) was varied from 6 to 15 Pa. (**d**) Normalized mean squared displacement (MSD) of all individual GFP spots when the in-plane stress was applied at 6, 9, 12 or 15 Pa at 0.3 Hz. No stress represents the spontaneous GFP movements in the absence of force application. (**e**) Rotation angles of the bead when applied stress was varied from 6 to 15 Pa. *P* value were 0.095, 0.36, 0.94 between 6 and 9 Pa, 9 and 12 Pa, 12 and 15 Pa respectively; *P* = 0.016 between 9 Pa and 15 Pa; *P* < 0.001 between 6 Pa and 12 Pa, 15 Pa. $^{*}P<0.05$; $^{***}P<0.001$. (**f**) Summarized data of the computed cell stiffness; ns=not significantly different. *P* value were 0.87, 0.69, 0.75 between 6 and 9 Pa, 9 and 12 Pa, 12 and 15 Pa respectively; *P* = 0.81, 0.55 between 6 Pa and 12 Pa, 15 Pa; *P* = 0.46 between 9 Pa and 15 Pa. Mean ± s.e.m.; n=42 cells, 35 independent experiments for (**d-f**). (**g**) Summarized data of chromatin deformation when applied stress was varied from 6 to 15 Pa. *P* value were 0.063, 0.79 between 6 and 9 Pa, 9 and 12 Pa; *P* = 0.019 for 12 and 15 Pa; *P* = 0.0019 between 6 and 12 Pa; *P* < 0.001 for 6 and 15 Pa, 9 and 15 Pa. Mean ± s.e.m.; n=126 GFP spots from 42 cells, 35 independent experiments; $^{*}P<0.05$; $^{**}P<0.01$; $^{***}P<0.001$. *P*-values were calculated and corrected using two-tailed Student's t-test and Bonferroni correction. Source data are provided as a Source Data file.

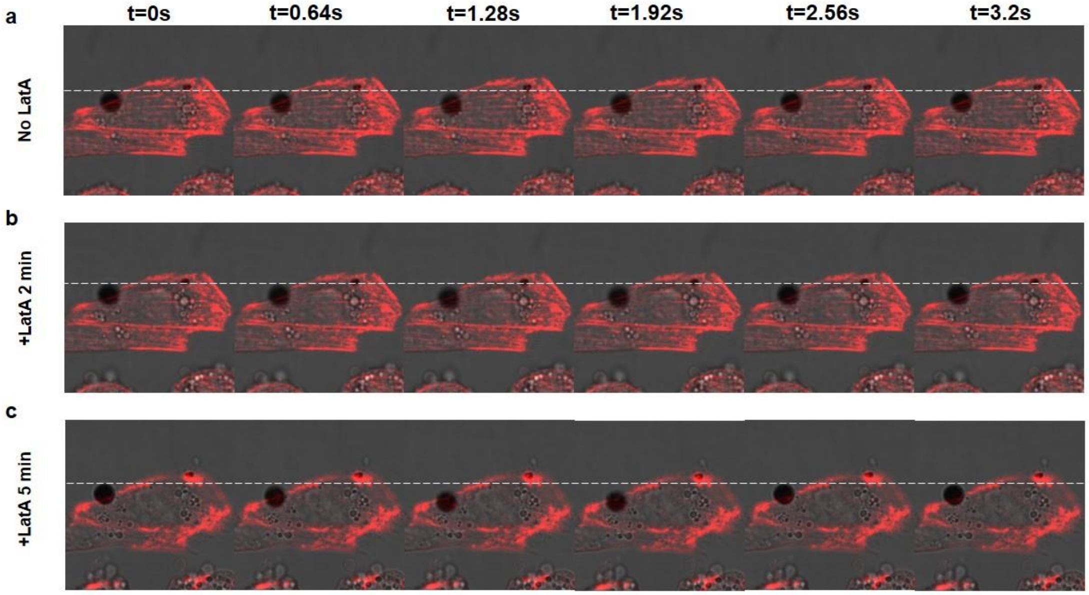

Supplementary Figure 3. Time course of stress fiber disruption in cells by Latrunculin A. F-actin was labelled by 0.1 percent（v/v）SiR-actin KIT (**red**) for 4 hr before the cells were treated with LatA. (**a**) Simultaneously recorded brightfield and fluorescence images of a representative cell before the cell was treated with LatA (No LatA). (**b**) Images of the same cell after treating with LatA for 2 min. (**c**) Images of the same cell after treating with LatA for 5 min. The stress was 15 Pa. The black dot was the magnetic bead attached on the apical surface of the cell. The white dash line is shown for visual aid. Note that the bead moved more after 5 min LatA when compared with those after 2 min LatA or at no LatA conditions, suggesting that the cell became softer when stress fibers were disrupted. From left to right: images were taken every 0.64 sec for one cycle of loading at 0.3 Hz.

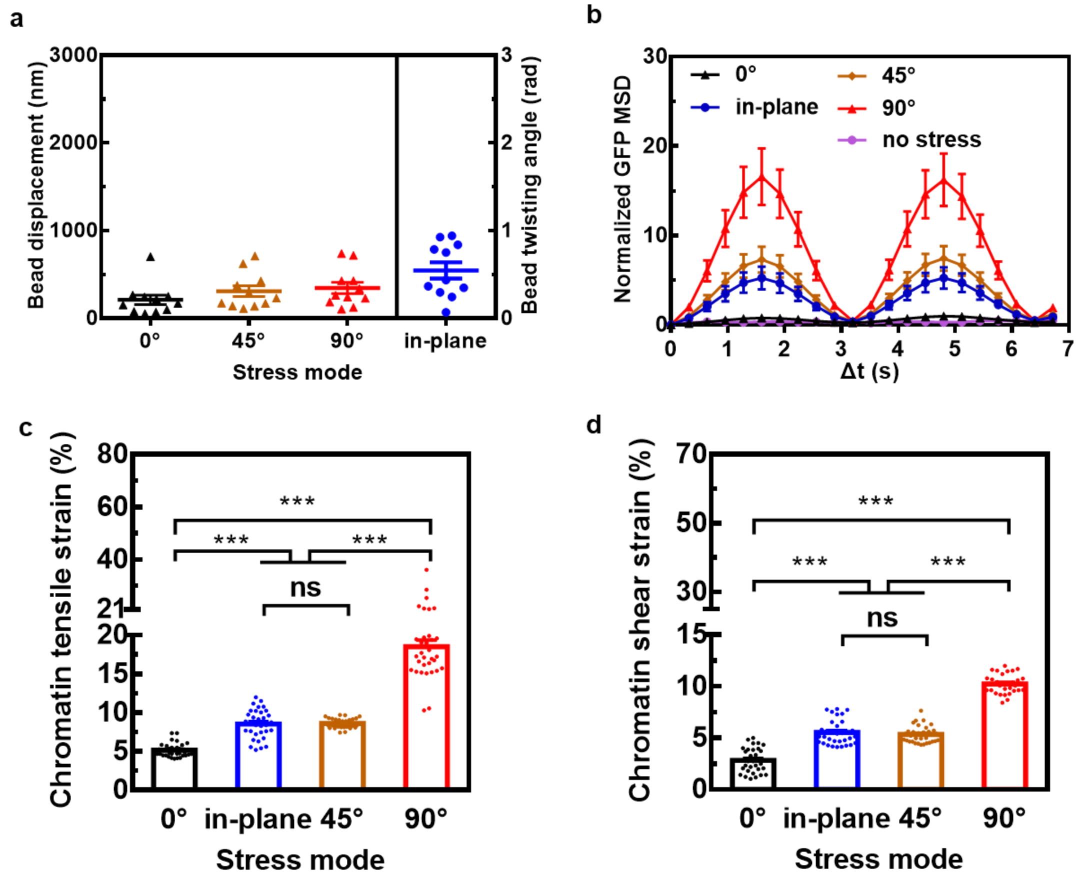

Supplementary Figure 4. Comparing in-plane stress and out-of-plane stress induced cell and chromatin deformation. (**a**) Peak displacements of the center of the magnetic bead (a measure of cell deformation) in response to an out-of-plane stress angle of 0 degree, 45 degrees, or 90 degrees or in-plane mode in the same cell. In all stress modes, the amplitudes of the sinusoidal magnetic fields were kept at 15 Pa and 0.3 Hz. n = 11 cells, 8 independent experiments. (**b**) Normalized mean squared displacement (MSD) of all individual GFP spots when the stress (15 Pa at 0.3 Hz) was applied at 0 degree, 45 degrees, or 90 degrees or in-plane. Data from five cycles of displacements were averaged in MSD curves. The MSD curves were normalized from 33 GFP spots from 11 cells, 8 independent experiments. (**c**) Tensile strains of the chromatin were computed from stress-induced chromatin deformation. $P = 0.85$ between in-plane and 45 degrees stress modes; $P < 0.001$ between each other different stress modes. (**d**) Shear strains were computed from the same chromatin deformation and were ~2 times less than tensile strains. $P = 0.42$ between in-plane and 45 degrees stress modes; $P < 0.001$ between each other different stress modes. For (a) – (d), Mean ± s.e.m.; n=11 cells, 8 independent experiments; ns = not significantly different; $^{***}P < 0.001$. For (**c-d**), P-values were calculated and corrected using two-tailed Student's t-test and Bonferroni correction. Source data are provided as a Source Data file.

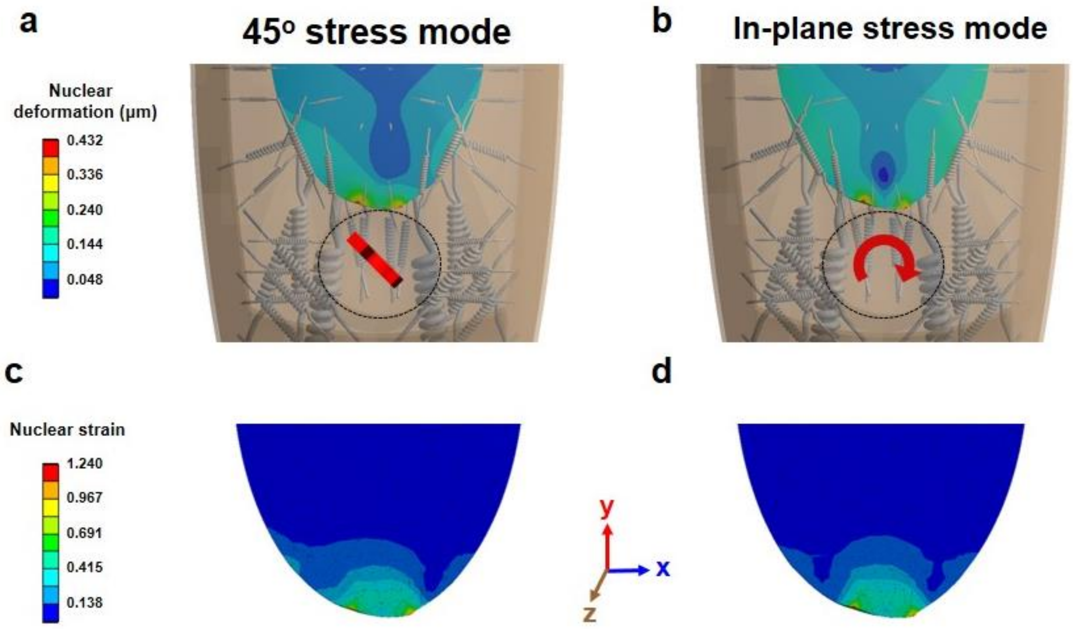

Supplementary Figure 5. Nuclear deformation and strain maps are similar under in-plane and 45 degrees stress modes in the discrete FEM. (**a**) Nuclear deformation under 45 degrees stress mode. (**b**) Nuclear deformation under in-plane stress mode. The color scale bar in (a) applies to (b). (**c**) Nuclear strain under 45 degrees stress mode. (**d**) Nuclear strain under in-plane stress mode. von Mises equivalent strain maps are shown. The color scale bar in (c) applies to (d). Note that the maximum strains are higher here than in Figure 7, possibly because stress fibers are modeled as spring elements here. The red line in (**a**) and the red arrow in (**b**) indicate the bead rolling direction; the black dotted line indicates the boundary of the magnetic bead on the cell surface.

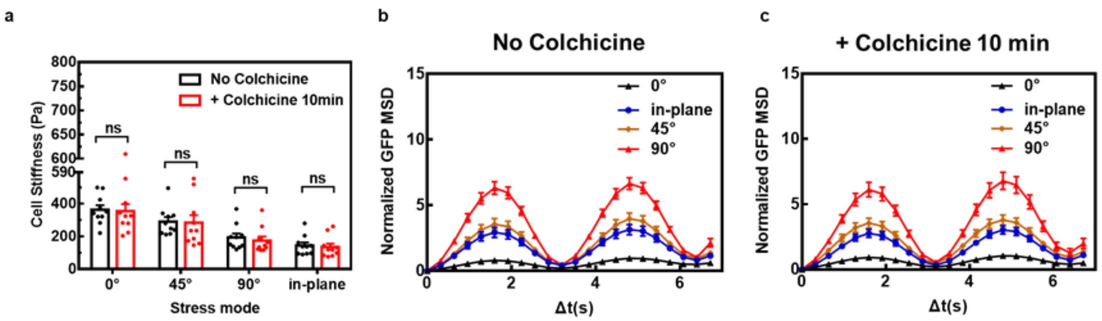

Supplementary Figure 6. **Microtubules do not contribute to cell stiffness and force propagation.** (**a**) Cell stiffness computed from different stress modes before adding colchicine and after adding colchicine for 10 min. P = 0.87, 0.89, 0.58 and 0.76 under 0 degree, 45 degrees, 90 degrees and in-plane stress modes respectively between No Colchicine and + Colchicine 10 min. P-values were calculated using two-tailed Student's t-test. Mean ± s.e.m.; n=10 cells, 7 independent experiments; ns = not significantly different. (**b, c**) Normalized mean squared displacement (MSD) of all individual GFP spots when the stress (15 Pa at 0.3 Hz) was applied at 0 degree, 45 degrees or 90 degrees out-of-plane mode or in-plane mode (**b**) before adding colchicine and (**c**) after adding colchicine. Data from five cycles of displacements were averaged in MSD curves. The MSD curves were normalized from 30 GFP spots from 10 cells, 7 independent experiments. Mean ± s.e.m. Extending duration of colchicine treatment to 20 or 30 min did not alter cell stiffness or chromatin deformation either. Source data are provided as a Source Data file.

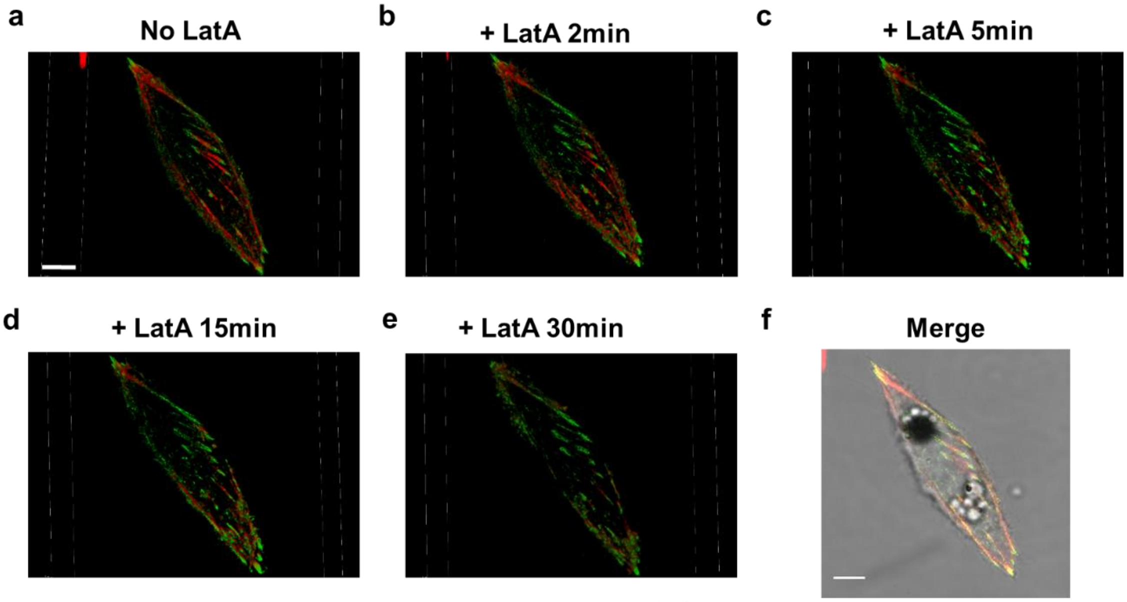

Supplementary Figure 7. Distributions of talin and F-actin in a cell at the cell basal surface before and after F-actin disruption. Images of a representative cell where talin was labelled by CellLight Talin-GFP (green) for 16 h before experiment and F-actin was labelled by SiR-actin KIT (red) for 4 h before the cell was treated with Latrunculin A (LatA, 1 μM). (**a**) Talin and F-actin distributions before LatA treatment. Scale bar, 5 μm. (**b-e**) After LatA treatment for 2, 5, 15, and 30 min. (**f**) Merged image of fluorescence and brightfield before LatA treatment. Scale bar, 5 μm. Note that focal adhesions (labeled with Talin-GFP) were minimally affected 5 min after LatA treatment and were still largely present by 30 min after most F-actin disappeared. Other cells exhibited similar responses.

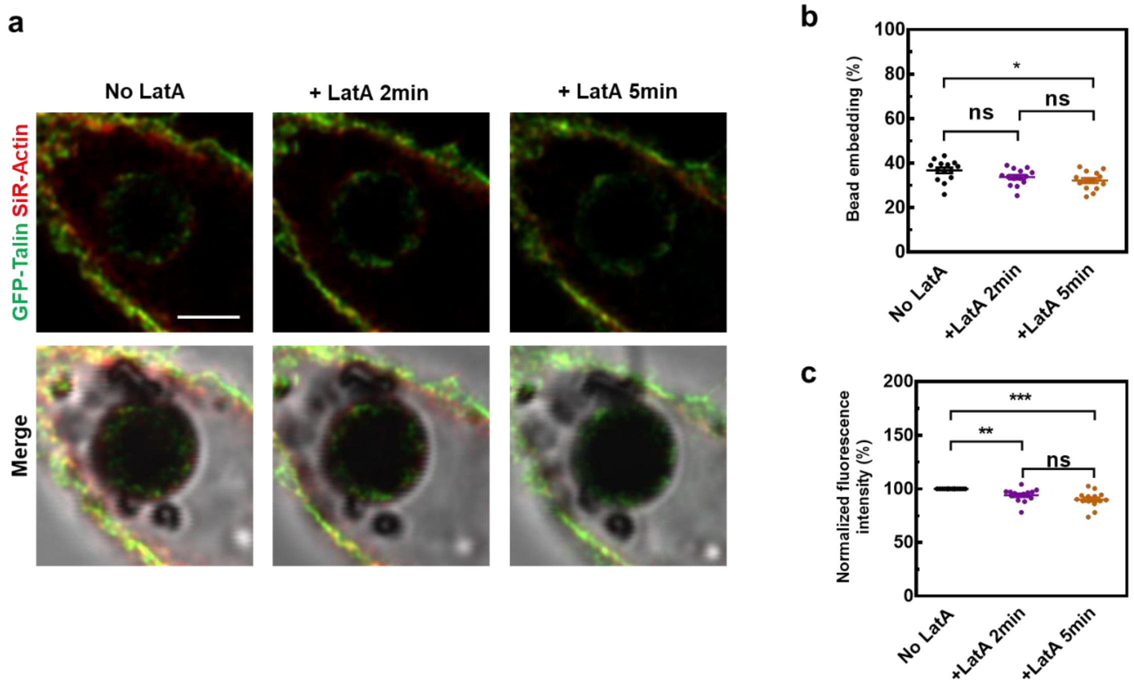

Supplementary Figure 8. RGD-bead cell contact area decreases slightly after 5-minute F-actin disruption. F-actin was disrupted with Latrunculin A (LatA, 1 μM) treatment. (**a**) A representative image of the bead-cell contact (the same cell as the one in Supplementary Figure 7). Top panel, talin-GFP and F-actin (green and red colors); Bottom panel, the merged image of brightfield and fluorescence. The large round black dot is the magnetic bead. Talin and F-actin fluorescence images were deconvoluted using Huygens software (Scientific Volume Imaging). Scale bar, 2.5 μm. (**b**) Summarized data of bead embedding before and after LatA treatment. Bead embedding (percentage of the bead surface area embedded into the cell membrane; 50 percent is when half of the bead is embedded) was calculated using the largest diameter of the x-y projected image of talin-GFP fluorescence as a measure of the largest bead-cell contact interface. P = 0.19 between No LatA and +LatA 2 min; P = 0.91 between +LatA 2 min and +LatA 5 min; P = 0.031 between No LatA and +LatA 5 min. (**c**) Quantification of changes in bead-cell contact area after F-actin disruption. Bead-cell contact area was estimated by measuring the x-y projected area times the average intensity of the largest fluorescence ring-like structure of talin-GFP surrounding the bead, which was an index of the bead-cell contact area, after taking into account of temporal controls without LatA of laser scanning induced photobleaching. Each contact area was normalized by the same bead before LatA treatment (No LatA). P = 0.0094 between No LatA and +LatA 2 min; P = 0.41 between +LatA 2 min and +LatA 5 min; P < 0.001 between No LatA and +LatA 5 min. For (**b**) and (**c**), mean ± s.e.m.; n=14 cells, 7 independent experiments. $^{*}P$ <0.05; $^{**}P$ <0.01, $^{***}P$ <0.001; ns=not significantly different. P-values were calculated and corrected using two-tailed Student's t-test and Bonferroni correction. Source data are provided as a Source Data file.

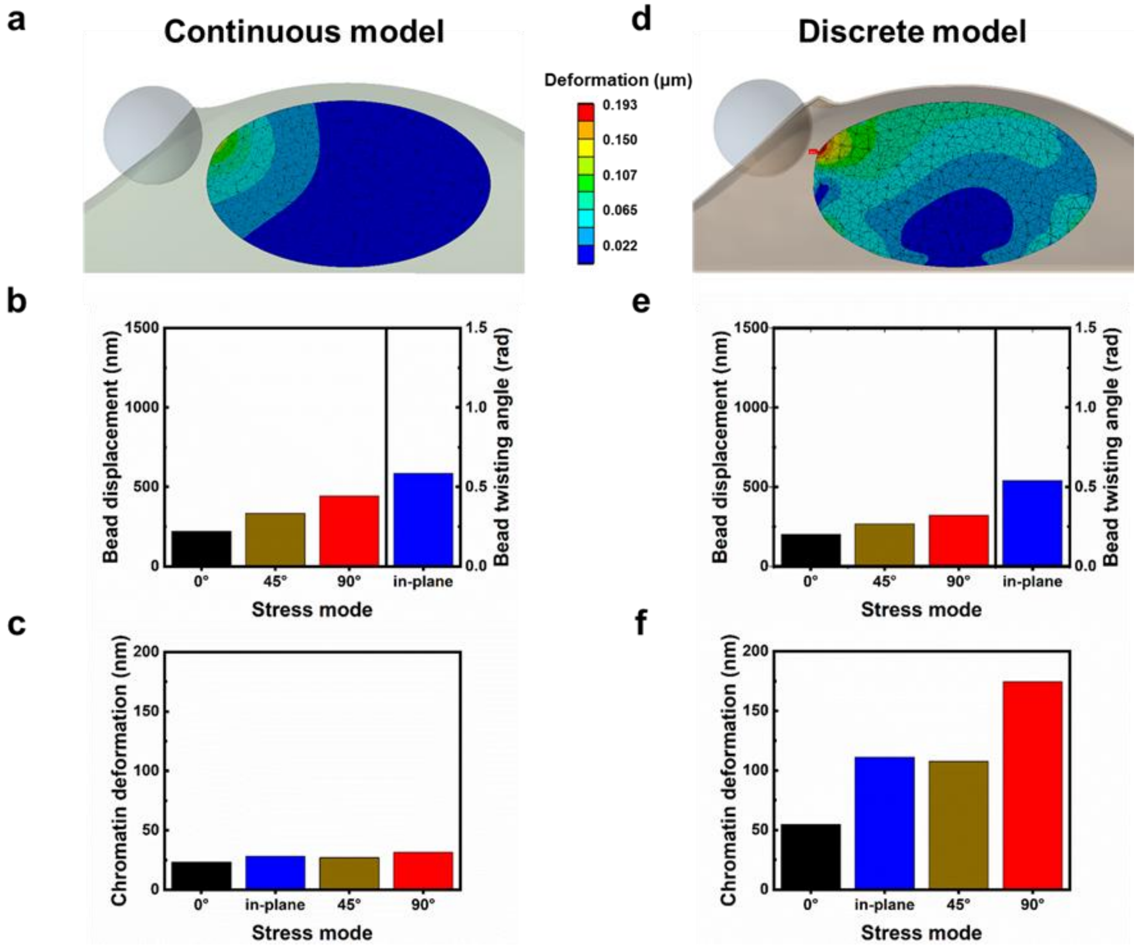

Supplementary Figure 9. Comparison of predictions between the discrete model and the continuous model. Continuous model **(a-c)**: (**a**) Nuclear deformation map. (**b**) Bead displacement or rotation angle (an index of cell deformation as a result of non-slip condition). (**c**) Chromatin deformation. Discrete model **(d-f)**: (**d**) Nuclear deformation map. (**e**) Bead displacement or rotation angle (an index of cell deformation). (**f**) Chromatin deformation. Please note that the cell and the bead are not drawn to scale in (**a**) and (**d**). The color scale bar applies to (a) and (d).

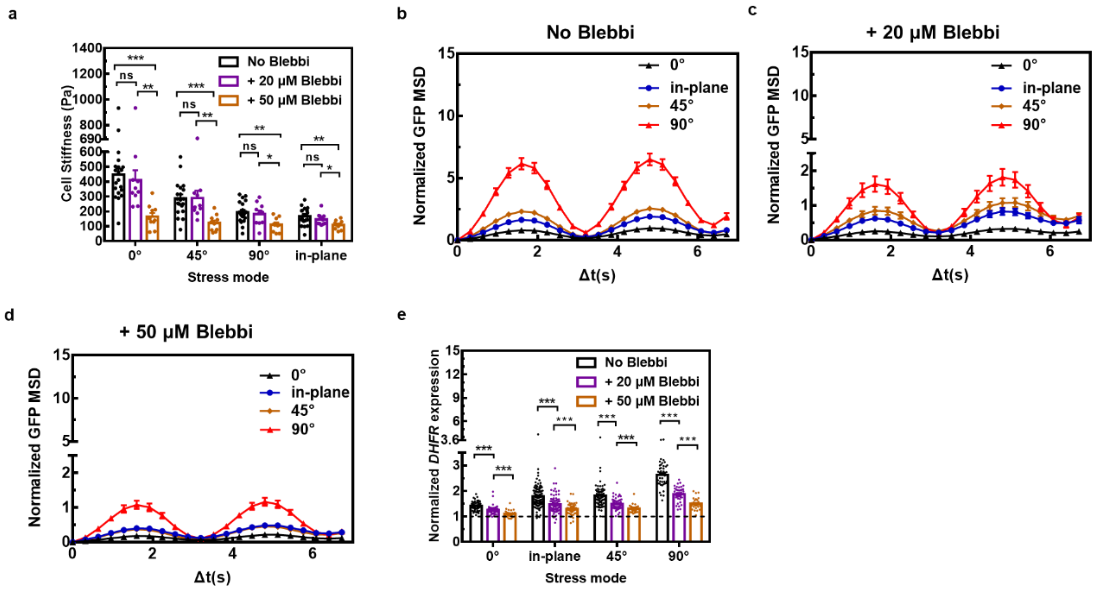

Supplementary Figure 10. Inhibiting myosin-II inhibits cell stiffness and chromatin deformation and gene upregulation. (**a**) Cell stiffness under different stress modes before adding Blebbistatin or after adding 20 μM Blebbistatin or 50 μM Blebbistatin for 30 min. Mean ± s.e.m.; n=20 cells, 12 independent experiments for No Blebbistatin; n=10 cells, 6 independent experiments for both + 20 μM Blebbistatin and + 50 μM Blebbistatin. *P* = 0.63, 0.97, 0.65 and 0.26 under 0 degree, 45 degrees, 90 degrees and in-plane stress modes respectively between No Blebbistatin and + 20 μM Blebbistatin; *P* = 0.0048, 0.0082, 0.022 and 0.031 under 0 degree, 45 degrees, 90 degrees and in-plane stress modes respectively between + 20 μM Blebbistatin and + 50 μM Blebbistatin; *P* < 0.001 for all stress modes respectively between No Blebbistatin and + 50 μM Blebbistatin. $^{*}P$ <0.05, $^{**}P$ <0.01, $^{***}P$ <0.001, ns=not significantly different. (**b-d**) Normalized mean squared displacement (MSD) of all individual GFP spots when the stress (15 Pa at 0.3 Hz) was applied at 0 degree, 45 degrees, or 90 degrees out-of-plane mode or in-plane mode before adding Blebbistatin (**b**) and after adding 20 μM Blebbistatin (**c**), 50 μM Blebbistatin (**d**) for 30 min. Data from five cycles of displacements were averaged in MSD curves. The MSD curves were normalized from 60 GFP spots from 20 cells, 12 independent experiments before adding Blebbistatin **(b)** and 30 GFP spots from 10 cells, 6 independent experiments for adding 20 μM Blebbistatin (**c**), 50 μM Blebbistatin (**d**) 30 min respectively. Mean ± s.e.m. (**e**) *DHFR* transcription inhibition with Blebbistatin treatment for both in-plane and out-of-plane modes. *P* < 0.001 between all the different conditions under each stress mode. Mean ± s.e.m.; 0 degree: n = 67, 53 and 32 cells; in-plane: n = 131, 80 and 61 cells; 45 degrees: n = 71, 67 and 30 cells; 90 degrees: n = 44, 45 and 34 cells for No Blebbistatin, 20 μM Blebbistatin, and 50 μM Blebbistatin, respectively; 3 independent experiments. $^{***}P$ <0.001. The dashed line indicated the *DHFR* gene expression levels of the cells in the same dishes without the beads (no applied stress). *P*-values were calculated using two-tailed Student's -test and Bonferroni correction Source data are provided as a Source Data file.

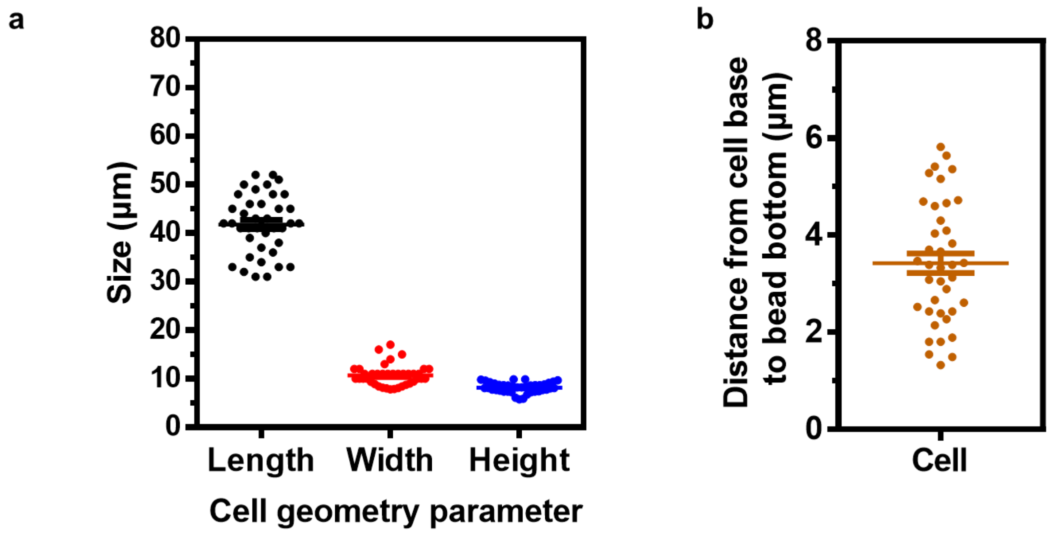

Supplementary Figure 11. Cell shape parameters. (**a**) Geometry parameters of CHO cells. Mean ± s.e.m.; n=39 cells, 3 independent experiments. (**b**) Distance from cell base to bead bottom. Mean ± s.e.m.; n=39 cells, 3 independent experiments. Source data are provided as a Source Data file.

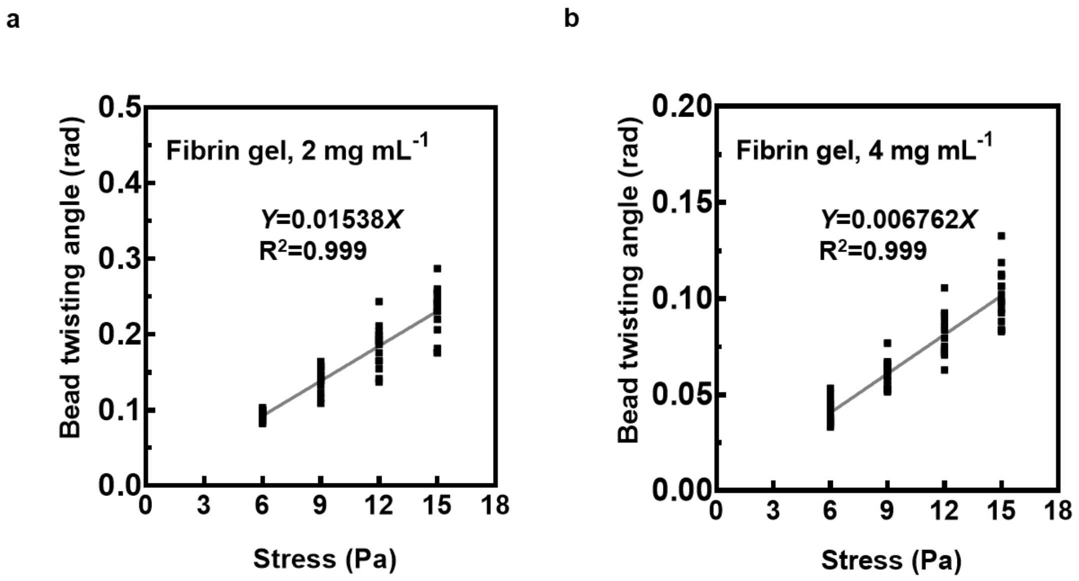

Supplementary Figure 12. Calibration of the magnetic bead assay in a uniform elastic gel. Individual magnetic beads were mixed in fibrinogen before fibrinogen activation by thrombin. The single beads were fully embedded in the uniform elastic fibrin gel of 2 mg mL^{-1} (**a**) or 4 mg mL^{-1} (**b**) and bead twisting angles (strain) were measured when stress was applied at various magnitudes with the in-plane stress mode at 0.3 Hz. N=15 beads; 3 independent experiments. The reciprocal of the slope was the shear elastic modulus, which was 65 Pa for (**a**) or 148 Pa for (**b**), consistent with the published values of 60 Pa or 140 Pa, respectively[1]. Source data are provided as a Source Data file.

Supplementary Table 1. Maximum and average normal strains and shear strains under different stress modes

	Normal strain		Shear strain		Normal strain /Shear strain	
	Maximum	Average	Maximum	Average	Maximum	Average
0°	0.415	0.165	0.172	0.074	2.41	2.23
45°	0.354	0.155	0.269	0.138	1.32	1.12
90°	0.318	0.138	0.373	0.179	0.85	0.77
In-plane	0.193	0.086	0.609	0.212	0.32	0.41

*FEM simulations of maximum and average normal strains and average shear strains at the cell cortex under different stress modes for the condition of the half angle of bead immersion ~70 degrees (~38 percent bead embedding). The absolute value of each strain was used to calculate the average strain under each condition. Source data are provided as a Source Data file.

Supplementary Table 2. Experimental and simulation parameters

	Experiment parameters	Simulation parameters
**Cell length**	41.79	40
**Cell width**	10.68	12
**Cell height**	8.19	8
**Bead diameter**	4.2	4
**Bead embedding**	36.3%	38.2%
**Cell base to bead bottom distance**	3.42	4

*Unit for all parameters is μm, except for bead embedding, for which it is the percentage of the bead surface area embedded into the cell membrane. Source data are provided as a Source Data file.

Supplementary Reference

1. Winer, J.P., Oake, S. & Janmey, P.A. Non-Linear Elasticity of Extracellular Matrices Enables Contractile Cells to Communicate Local Position and Orientation. *PLoS One.* **4**, e6382 (2009).
